# Bi-valent polysaccharides of Vi capsular and O9 O-antigen in attenuated *Salmonella* Typhimurium induce strong immune responses against these two antigens

**DOI:** 10.1038/s41541-017-0041-5

**Published:** 2018-01-09

**Authors:** Pei Li, Qing Liu, Hongyan Luo, Kang Liang, Yue Han, Kenneth L. Roland, Roy Curtiss, Qingke Kong

**Affiliations:** 1 Institute of Preventive Veterinary Medicine, College of Veterinary Medicine, Sichuan Agricultural University, Chengdu, Sichuan 611130 China; 2 Center for Infectious Diseases and Vaccinology, The Biodesign Institute, Arizona State University, Tempe, AZ 85287 USA; 3 College of Animal Science and Technology, Southwest University, Chongqing, 400715 China; 4 Department of Infectious Diseases and Immunology, University of Florida, Gainesville, FL 32608 USA

## Abstract

*Salmonella* Typhi is the causative agent of typhoid fever in humans, responsible for approximately 21 million infections and 222,000 deaths globally each year. The current licensed vaccines provide moderate protection to recipients aged >2 years. Prior work on typhoid vaccines has focused on injectable Vi capsular polysaccharide or Vi–protein conjugates and live, oral attenuated *S*. Typhi vaccines to induce humoral anti-Vi antibodies, while the value and importance of anti-O9 antibodies is less well established. In this study, we constructed a *S*. Typhimurium strain that synthesizes Vi capsular antigen in vivo and produces the immunodominant O9-antigen polysaccharide instead of its native O4-antigen. The live recombinant attenuated *S*. Typhimurium mutants were effective in stimulating anti-Vi and anti-O9 antibodies in a mouse model, and the surface Vi capsular expression did not affect the immune responses against the O9 O-antigen polysaccharide. Moreover, the resulting anti-Vi and anti-O9 antibodies were effective at killing *S*. Typhi and other *Salmonella* spp. expressing Vi or O9 antigen polysaccharides and provided efficient protection against lethal challenge by *S*. Typhimurium and *S*. Enteritidis. Our work highlights the strategy of developing live attenuated *S*. Typhimurium vaccines to prevent typhoid fever by targeting the both Vi capsular and O9 O-polysaccharide antigens simultaneously.

## Typhoid fever: Turning Salmonella bacteria against each other

An attenuated strain of modified *Salmonella* Typhimurium bacteria could answer calls for a more effective typhoid fever vaccine. Current vaccines against typhoid-causing *Salmonella* Typhi are only moderately effective and potentially ineffective in children under 5 years. Qingke Kong and Roy Curtiss, leading a team of US and Chinese researchers, developed an attenuated version of the less-pathogenic *S*. Typhimurium that, when orally dosed in mice, expresses bacterial sugar-chain molecules known to elicit a strong immune response. In an in vitro assay, the antibodies produced by the mice in response to these molecules killed *S*. Typhi and related *Salmonella* bacteria with similar surface molecules, indicating a potential cross-protective ability. Further research would reveal whether this two-pronged live vaccine has the potential to protect in vivo, in live animals and in humans.

## Introduction

Typhoid fever is a systemic infection in humans caused by *Salmonella enterica* subsp. *enterica* serovar Typhi (*S*. Typhi), with symptoms of fever, chills, respiratory distress, and abdominal pain, which are often non-specific and clinically indistinguishable from other febrile illnesses.^1^ Although the first inactivated *S*. Typhi vaccine was licensed >100 years ago, typhoid fever remains a major public health concern with high mortality and morbidity worldwide. The global burden of typhoid fever estimated was approximately 21 million infections and 222,000 deaths annually.^2^ Typhoid fever outbreaks are frequently reported in Sub-Saharan Africa, Southeast Asia, and other developing countries, with infants, children, and adolescents being the most commonly infected. In developed countries, most typhoid fever cases occur among travelers returning from typhoid-endemic areas.^3^ Typhoid fever is predominantly associated with poor sanitation and asymptomatic carriers are often a source of contamination in food and water. The emergence of antibiotic resistance in clinical isolates of *S*. Typhi has resulted in typhoid fever being increasingly difficult to treat.^4^ In the short term, vaccination is the most effective and economic method to prevent this disease.

The World Health Organization (WHO) currently recommends two vaccines for controlling typhoid fever outbreaks, an injectable purified Vi polysaccharide vaccine and a live attenuated oral Ty21a vaccine in capsule formulation. However, both vaccines are only moderately protective (50–70%), and their efficacies in preschool-aged children (<5 years of age) are either not acceptable or unknown.^5^ Unfortunately, there is a high incidence of typhoid fever in children aged <2 years in developing countries.^6^ Therefore, new vaccines with higher protective efficacy and immune responses in younger age groups are urgently needed.

Current typhoid vaccine development focuses primarily on injectable Vi-protein conjugates and oral live attenuated *S*. Typhi. Compared to Vi-conjugate vaccines, live attenuated vaccines offer a needle-free alternative, inducing strong humoral and mucosal immune responses, T-cell responses, and long-term T-cell memory. The idea of modifying the expression of one or more of the *tviABCDE* genes required for Vi synthesis has been explored. In strain CVD909, expression of *tviABCDE* was placed under transcriptional control of the P_tac_ promoter, resulting in constitutive synthesis of Vi capsular polysaccharide.^7,8^ However, none of these live vaccine candidates were effective at stimulating strong humoral anti-Vi serum responses.^8,9^ Another approach to modifying *tviABCDE* expression has been to replace the native P_tviA_ promoter with the in vivo-inducible P_ssaG_ promoter from the *Salmonella* pathogenicity island 2 (SPI-2) gene *ssaG* of *S*. Typhimurium. A virulent *S*. Typhimurium strain carrying this construct elicited high levels of anti-Vi serum IgG after a single oral administration in mice.^10^

Antibodies against Vi and O9 O-antigen polysaccharide are each capable of directing complement-mediated killing of *S*. Typhi, though killing by anti-O9 antibodies is dependent on the level of Vi antigen expression.^11^ The protection conferred by the Food and Drug Administration-licensed typhoid vaccine Ty21a (Vi^−^) is believed to be due in part to increased serum and mucosal antibodies against *S*. Typhi lipopolysaccharides (LPS),^12^ while high anti-LPS titers were noted in individuals vaccinated with the experimental strain Ty800.^13^ In light of the importance of both antigens, we constructed an attenuated *S*. Typhimurium-vectored typhoid vaccine engineered to produce both Vi and O9 polysaccharides.

The Vi capsular polysaccharide of *S*. Typhi is a linear homopolymer of α-1,4-linked *N*-acetylgalactosaminuronate (Gal*N*AcA) with 60–70% *O*-acetylation at the monomeric C-3 position.^14^ The lack of free hydroxyl groups in the Vi capsular inhibits C3 fixation and prevents complement deposition on the *S*. Typhi surface.^15^ Vi capsular synthesis is regulated by several regulatory systems, including *ompR*-*envZ*,^16^ *rscB*-*rscC*,^17^ and RpoS, an alternative sigma factor,^18^ and the former two regulators will upregulate Vi synthesis upon encountering a condition from high osmolarity to low osmolarity, while RpoS is involved in fine-tuning the synthesis of Vi capsular polysaccharide during this stage. The genes required for the biosynthesis of the Vi capsular is exclusively found in Vi-expressing strains. The *viaB* locus, residing on a 134-kb DNA island termed *Salmonella* pathogenicity island 7 (SPI-7), is composed of 10 genes involved in regulation (*tviA*), biosynthesis (*tviBCDE*), and export (*vexABCDE*) of the Vi capsular polysaccharide.^19^ Tight regulation of Vi capsular production is coordinated primarily via the TviA protein, a positive regulator of Vi capsular biosynthesis and a negative regulator of flagellar gene expression and secretion of SPI-1 effectors.^20,21^ The *tviA* promoter (P_tviA_) is repressed under the high-osmolarity conditions in the intestinal lumen but is rapidly induced in the low-osmolarity environment present in tissues.^22,23^ The VexE protein is required for Vi capsular anchoring in the outer membrane, and deletion of *vexE* leads to the extracellular release of the Vi capsular.^24^ Vi capsular biosynthesis initiates from the inner plasma membrane, and mutations in genes encoding the export machinery (*vexABCD*) results in intracellular Vi capsular accumulation.^19^

The O-antigens of *S*. Typhimurium (B1 group, immunodominant O4 serotype) and *S*. Typhi (D1 group, immunodominant O9 serotype) share a common trisaccharide backbone of 2)-α-Man(1→4)-α-Rha-(1→3)-α-Gal-(1→.^25^ The unique dideoxyhexose sugars abequose and tyvelose contribute to O4 or O9 serogroup specificity, respectively. The gene cluster accounting for O-antigen polysaccharide synthesis of *Salmonella* O4 and O9 is located between *galF* and *gnd* in the chromosome.^26^ The main differences between the gene clusters are the genes responsible for synthesis of these two unique dideoxyhexose sugars.^27^ We have shown previously that *S*. Typhimurium with *abe* gene replacement with *prt-tyv*_D1_ from *S*. Enteritidis could convert the O4 serotype to O9.^28^

In this study, we replaced the native *tviA* promoter (P_tviA_) in the *viaB* locus with *ssaG* gene promoter (P_ssaG_)^10^ and then introduced this in vivo-regulated *viaB* locus into an O9 serotype-converted live attenuated *S*. Typhimurium vaccine to stimulate production of both anti-Vi capsular and anti-O9 O-antigen polysaccharide antibodies. Our results showed that the live recombinant attenuated *S*. Typhimurium mutants were effective in stimulating anti-Vi and anti-O9 antibodies in a mouse model and that the resulting anti-Vi and anti-O9 antibodies were effective at killing *S*. Typhi and other *Salmonella* spp. expressing Vi or O9 antigen polysaccharides.

## Results

### Construction of the live attenuated *S*. Typhimurium vaccine candidates

**Fig. 1:**
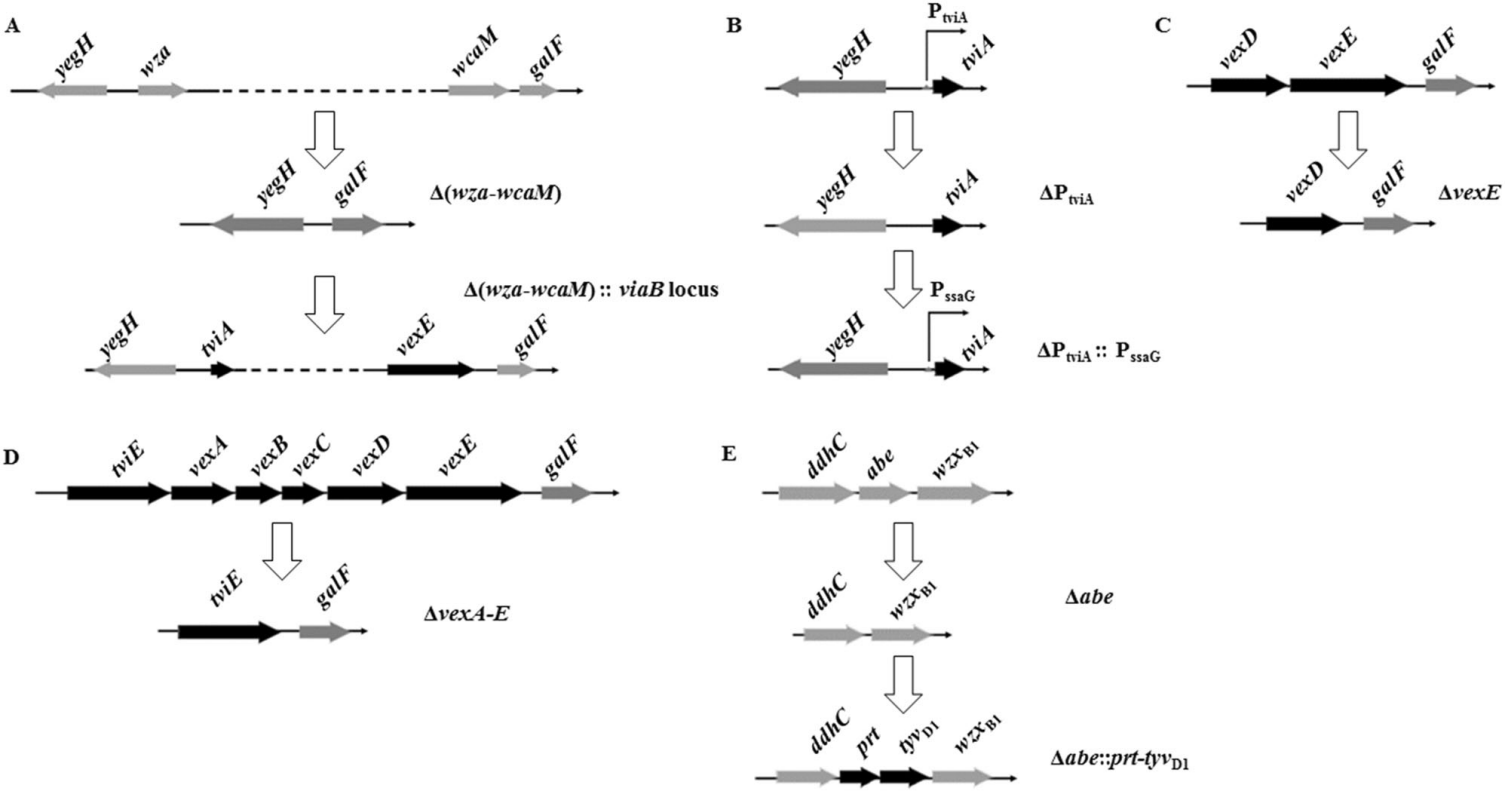
Deletion–insertion mutations in *S*. Typhimurium. **a** The entire colanic acid (CA) operon was replaced with an intact *viaB* locus from *S*. Typhi. **b** The native P_tviA_ promoter in the *viaB* locus was replaced by the P_ssaG_ promoter. **c** Deletion of the* vexE* gene in the *viaB* locus. **d** Deletion of the *vexA-E* genes in the *viaB* locus. **e** The allelic *abe* gene was replaced by *prt*-*tyv*_D1_ from *S*. Enteritidis, resulting in O9 O-polysaccharide expression in *S*. Typhimurium

To obtain effective live vaccines against *S*. Typhi infection, we constructed a *S*. Typhimurium mutant capable of producing two protective antigens, the Vi capsular and the O9 O-antigen polysaccharide (Supplementary Fig. S1). To construct a Vi^+^ strain, the entire colanic acid (CA) operon was deleted from *S*. Typhimurium and replaced with a functional *viaB* locus from *S*. Typhi, resulting in *S*. Typhimurium mutant strain S1123 (O4, Vi^+^) (Fig. 1a). To avoid downregulation of Vi capsular gene expression in antigen-presenting cells, we replaced the native P_tviA_ promoter with the intracellular-inducible SPI-2 promoter, P_ssaG_ (Fig. 1b),^10,29^ resulting in S1137 (O4, Vi^+^, ΔP_tviA_::P_ssaG_). The promoter-modified *viaB* locus was then introduced into the O9 serotype-converted *S*. Typhimurium mutant S1032 to generate S1151 (O9, Vi^+^, ΔP_tviA_::P_ssaG_). The O9 serotype conversion in *S*. Typhimurium was achieved by replacing the *abe* gene with *prt*-*tyv*_D1_ (Fig. 1e). Because the Vi capsular polysaccharide may mask the O9 O-antigen polysaccharide in *S*. Typhimurium, we constructed two additional strains that resulted in Vi accumulation in either the culture supernatant or retained in the cytoplasm. Strain S1159 (O9, Vi^+^, ΔP_tviA_::P_ssaG_ Δ*vexE*), which releases Vi polysaccharide into the culture supernatant, was constructed by deleting *vexE* (Fig. 1c).^19,24^ Strain S1160 was engineered to accumulate Vi intracellularly by deleting the *vexA-E* genes responsible for Vi export (Fig. 1d).^19^ These genetic modifications were introduced into the live attenuated *S*. Typhimurium vaccine S738 (Δ*crp* Δ*cya*) to evaluate their impact on immunogenicity and protective efficacy.^30,31^ In summary, the newly constructed live recombinant attenuated vaccine candidates were S1148 (O4, Vi^+^, ΔP_tviA_::P_ssaG_ Δ*cya* Δ*crp*), S1163 (O9, Vi^+^, ΔP_tviA_::P_ssaG_ Δ*cya* Δ*crp*), S1167 (O9, Vi^+^, ΔP_tviA_::P_ssaG_ Δ*vexE* Δ*crp* Δ*cya*), and S1168 (O9, Vi^+^, ΔP_tviA_::P_ssaG_ Δ*vexA-E* Δ*crp* Δ*cya*).

### Sustainable Vi capsular expression in O9 serotype-converted *S*. Typhimurium

**Fig. 2:**
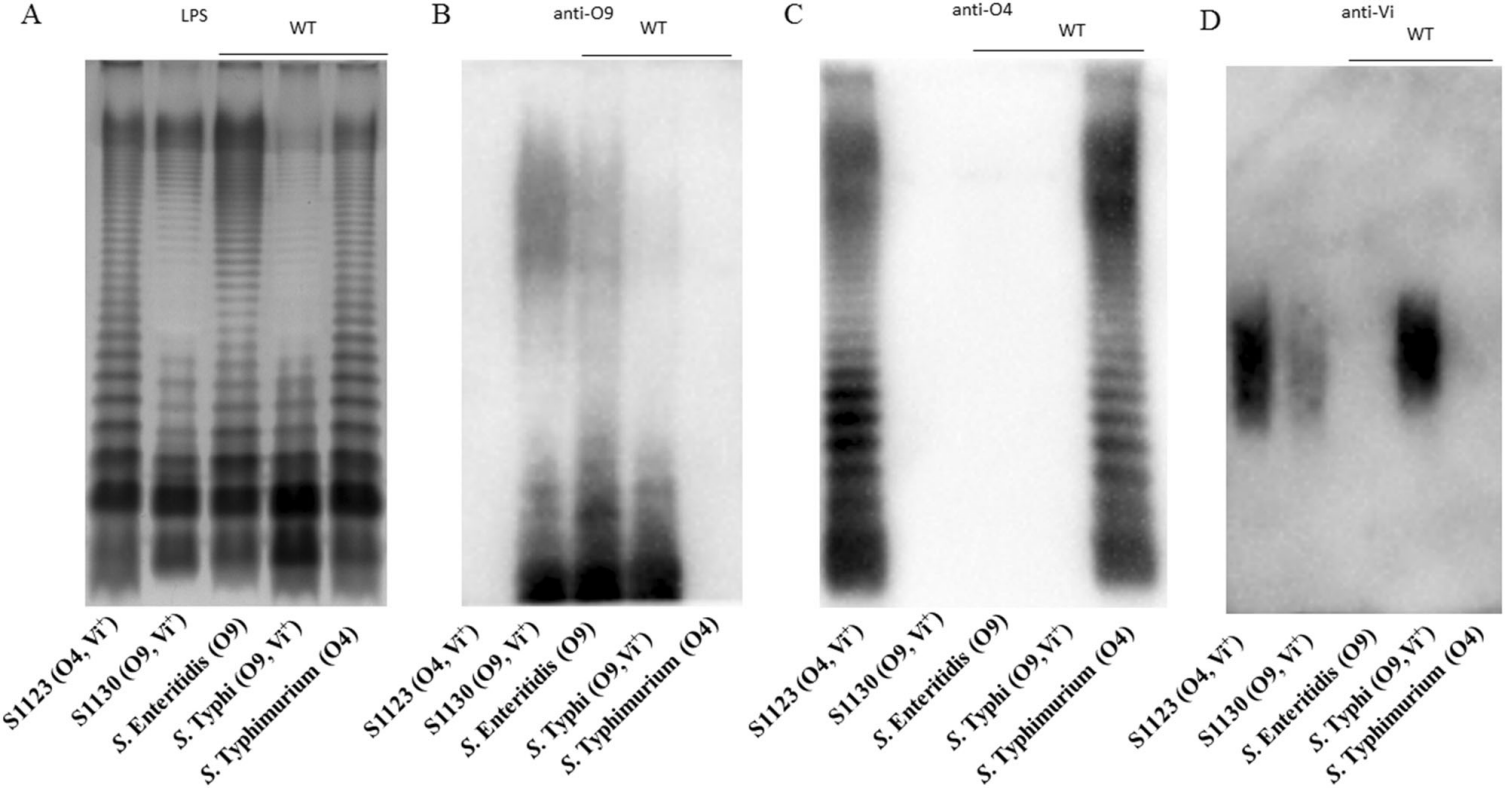
Outer membrane polysaccharide silver staining and western blotting. Samples derived from the same experiment and gel/blots were processed in parallel. **a** LPS profiles of S1123 (O4, Vi^+^), S1130 (O9, Vi^+^), and wild-type strains *S*. Enteritidis, *S*. Typhi, and *S*. Typhimurium. **b** Western blots probed with anti-O9 (group D1) single-factor antisera. **c** Western blots probed with anti-O4 (group B1) single-factor antisera. **d** Western blots probed with anti-Vi antisera

O9 O-antigen polysaccharide and Vi capsular production was visualized by silver staining and confirmed by western blotting (Fig. 2). The wild-type *S*. Typhimurium and *S*. Enteritidis strains display long O-antigen polysaccharides containing 20–100 repeating units (Fig. 2a), while most of the *S*. Typhi O-antigen polysaccharides are short.^32^ Introduction of the Δ(*wza*-*wcaM*)::*viaB* and Δ*abe*::*prt*-*tyv*_D1_ mutations did not influence LPS length, with all *S*. Typhimurium strains exhibiting the expected long-chain O-antigen polysaccharide (Fig. 2a). Western blotting results showed that the O-antigen produced by *S*. Typhimurium strain S1130 reacted with anti-O9 antisera and no longer reacted with anti-O4 antisera, as expected (Fig. 2b, c).

**Fig. 3:**
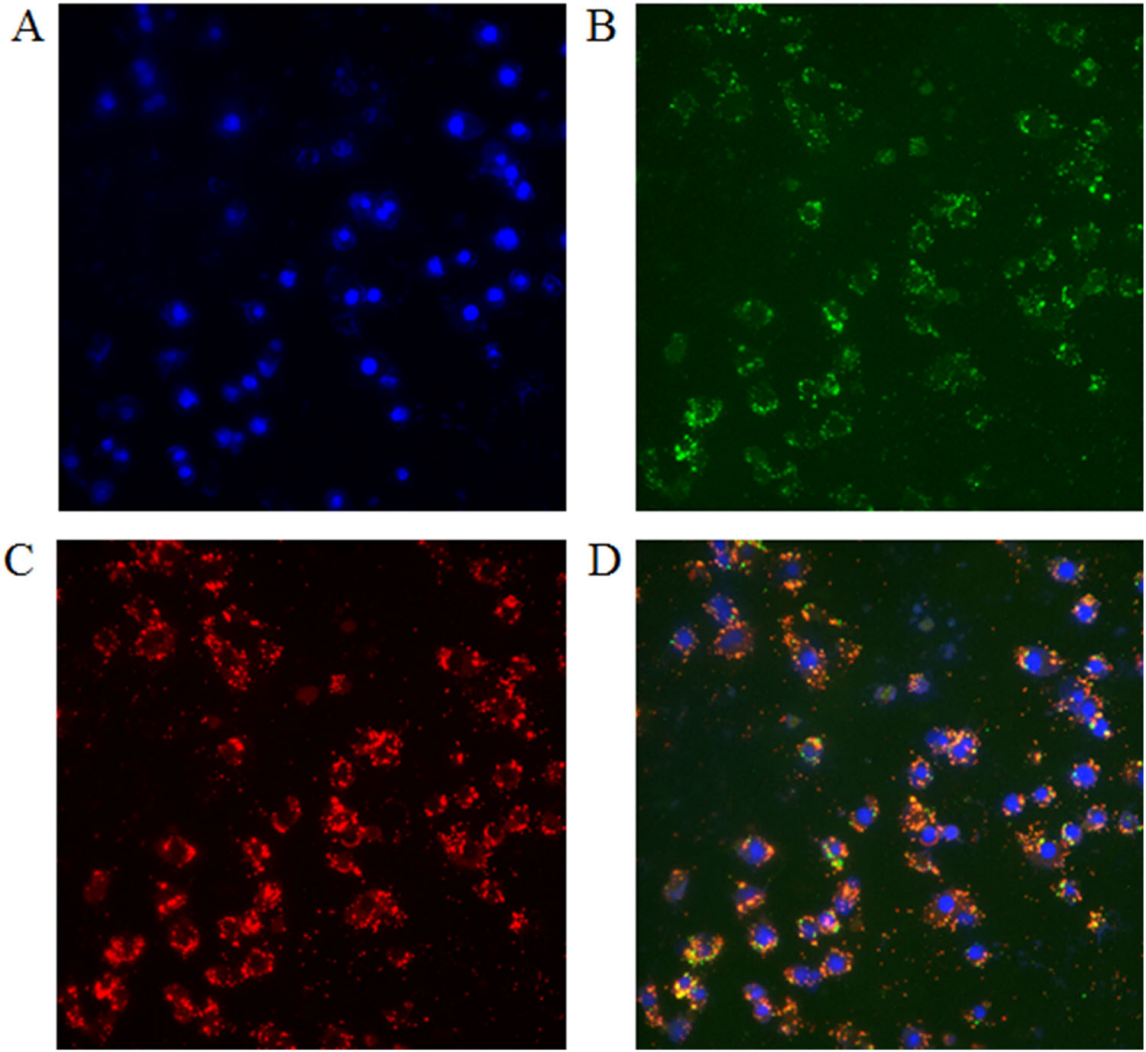
In vitro intracellular Vi capsular expression in *S*. Typhimurium. Production of Vi capsular in S1157 (O9, Vi^+^, ΔP_tviA_::P_ssaG_) was evaluated in RAW264.7 macrophages in vitro. The panels represented different channels: **a** blue, nucleic acid stained by DAPI; **b** green, S1157 carrying a GFP^+^ expression plasmid (pYA4518); **c** red, Vi polysaccharides probed with anti-Vi antisera, and **d** a merged image. Images were taken at 10 × 40 magnification

Production of Vi capsular by strains S1123 (O4, Vi^+^) and S1130 (O9, Vi^+^) mutants was confirmed by western blotting using anti-Vi antisera (Fig. 2d). When *S*. Typhi invades the mucosal epithelium or phagocytic cells, Vi capsule is produced to evade innate immune responses.^33,34^ Despite these findings, there is evidence that the production of Vi antigen is downregulated in the phagosomal compartments in vivo,^35^ with little or no Vi production in the spleen and liver.^10^ However, placing *viaB* under transcriptional control of the SPI-2 P_ssaG_ promoter allows sustained Vi production within the *Salmonella*-containing vacuole in macrophages.^10,29^ We infected RAW264.7 cells with S1151 (O9, Vi^+^, ΔP_tviA_::P_ssaG_) to confirm that Vi capsule is produced intracellularly. As depicted in Fig. 3, replacement of the natural P_tviA_ promoter with the intra-macrophage-inducible P_ssaG_ promoter resulted in Vi polysaccharide production within macrophages.^10^ This is consistent with previous results showing that the *ssaG* promoter is induced 400-fold in macrophages.^36^

### Characterization of the *S*. Typhimurium mutants in vitro

All mutant strains were transducible with phage P22 (Supplementary Table S2), indicating that the converted O-polysaccharide was recognized by P22 and that in vitro grown cells did not produce enough Vi to block P22 infection. Susceptibility to deoxycholate did not differ among strains, and differences in sensitivity to polymyxin B among the recombinant strains were increased by two-fold (Supplementary Table S2). All strains had growth rates similar to wild-type strain S100, except strain S1160 (O9, Vi^+^, ΔP_tviA_::P_ssaG_ Δ*vexA-E*), which showed a moderate decrease in growth rate (Supplementary Fig. S2). Swimming motility for each mutant was similar for all strains except strain S1160 (O9, Vi^+^, ΔP_tviA_::P_ssaG_ Δ*vexA-E*), which exhibited a significant decrease in swimming motility compared to wild-type strain S100, though it can still be considered to be motile (Supplementary Table S2).

As successful live attenuated *Salmonella* vaccines must attach and invade host gastrointestinal epithelial cells, we evaluated the ability of our Δ*cya* Δ*crp* vaccine strains to interact with Hep-2 cells. Compared to the parental strain S1114 (O4, Δ*crp* Δ*cya*), all strains attached equally well, except strain S1167, which produces Vi intracellularly. This strain exhibited a significantly lower rate of attachment (Supplementary Fig. S3). In addition, we observed a significantly lower rate of invasion for all strains carrying *viaB*.

### Virulence and colonization of the *S*. Typhimurium mutants in BALB/c mice

The wild-type strains *S*. Typhimurium S100 and *S*. Enteritidis S246 are highly virulent in a murine model with an LD_50_ value of approximately 1 × 10^5^ colony-forming units (CFU). The LD_50_ values of the S100 derivatives S1137 (O4, Vi^+^, ΔP_tviA_::P_ssaG_), S1151 (O9, Vi^+^, ΔP_tviA_::P_ssaG_), S1159 (O9, Vi^+^, ΔP_tviA_::P_ssaG_ Δ*vexE*), and S1160 (O9, Vi^+^, ΔP_tviA_::P_ssaG_ Δ*vexA-E*) were approximately 10^8^ CFU (Supplementary Table S2), showing attenuation of approximately three orders of magnitude.

We next evaluated colonization of host tissues by the Δ*cya* Δ*crp* derivatives of each strain. Mice were orally inoculated with approximately 1 × 10^9^ CFU of each strain. Peyer’s patches, spleens, and livers were harvested 4 and 8 days later. No significant differences were detected in colonization of the Peyer’s patches among the mutants. While all strains colonized spleen and liver equally well on day 4, by day 8 we recovered significantly fewer CFUs of strains S1148, S1163, S1167, and S1168 as compared to the parental S1114. No deaths occurred during this period (Supplementary Fig. S4).

### Immune responses induced by live attenuated *S*. Typhimurium vaccines

**Fig. 4:**
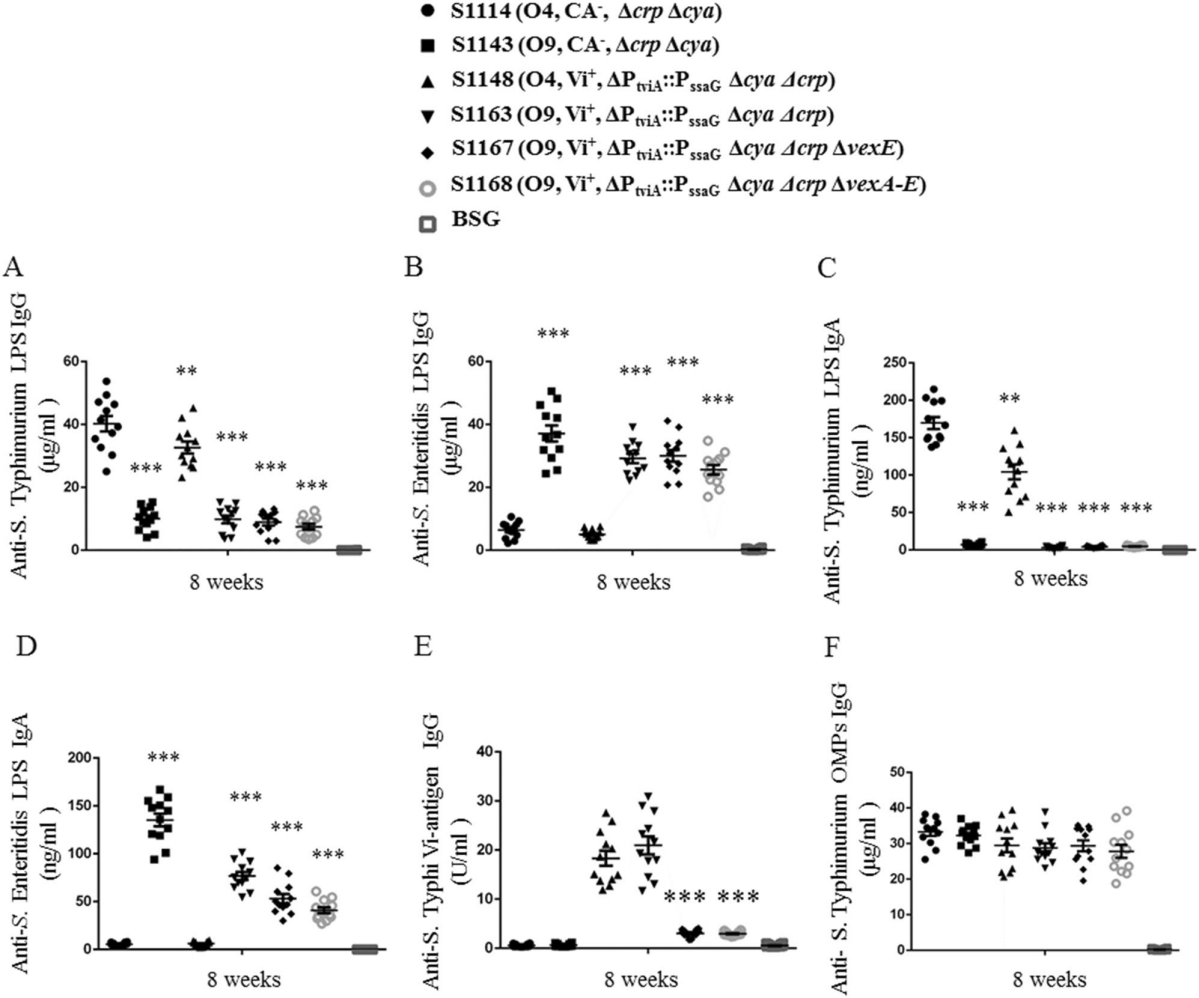
Antibody responses in mice sera and vaginal secretions determined by ELISA. Anti-*S*. Typhimurium and anti-*S*. Enteritidis LPS antibody concentrations in the vaccinated mice sera (**a**, **b**) and vaginal secretions (**c**, **d**). **a** Anti-*S*. Typhimurium LPS serum IgG levels in immunized mice. Responses that differed from the results in the S1114 (O4, CA^−^, Δ*crp* Δ*cya*) group are noted by asterisks (**P < 0.01; ***P < 0.001). **b** The anti-*S*. Enteritidis LPS serum IgG levels. Responses that differed from the results in the S1114 (O4, CA^−^, Δ*crp* Δ*cya*) group are noted by asterisks (***P < 0.001). **c** Anti-*S*. Typhimurium LPS mucosal IgA levels in immunized mice. Responses that differed from the results in the S1114 (O4, CA^−^, Δ*crp* Δ*cya*) group are noted by asterisks (**P < 0.01; ***P < 0.001). **d** Anti-*S*. Enteritidis LPS IgA levels in immunized mice. Responses that differed from the results in the S1114 (O4, CA^-^, Δ*crp* Δ*cya*) group are noted by asterisks (***P < 0.001). **e** Serum IgG responses against *S*. Typhi Vi capsule. No significant difference between the sera samples vaccinated by S1148 (O4, Vi^+^, ΔP_tviA_::P_ssaG_ Δ*cya* Δ*crp*) and S1163 (O9, Vi^+^, ΔP_tviA_::P_ssaG_ Δ*cya* Δ*crp*) were detected. A significantly lower level of anti-Vi IgG response was observed in the S1167 (O9, Vi^+^, ΔP_tviA_::P_ssaG_ Δ*vexE* Δ*crp* Δ*cya*) and S1168 (O9, Vi^+^, ΔP_tviA_::P_ssaG_ Δ*vexA-E* Δ*crp* Δ*cya*) strains than in the S1148 strain (***P < 0.001). **f** Anti-*S*. Typhimurium OMPs IgG levels. No significant differences in responses between groups were detected. Antibody concentrations were calculated using a standard curve and all the measured sample concentrations were within the standard curve range. The error bars represent the standard errors of the means as calculated by the GraphPad Prism software

To assess the immunogenicity of our vaccine candidates, mice were orally inoculated with approximately 1 × 10^9^ CFU of each vaccine strain on day 0 and boosted on day 28 with the same dose. Anti-*S*. Typhimurium LPS (O4), anti-*S*. Enteritidis LPS (O9), anti-*S*. Typhi Vi capsular, and anti-*S*. Typhimurium outer membrane protein (OMP) antibodies in mice sera and vaginal secretions were measured on day 56 (Fig. 4 and Supplementary Fig. S5). The mice vaccinated with S1114 (O4, CA^−^, Δ*crp* Δ*cya*) mounted a significantly higher anti-*S*. Typhimurium LPS immune response than S1148 (O4, Vi^+^, ΔP_tviA_::P_ssaG_ Δ*cya* Δ*crp*) and the O9 serotype vaccines (Fig. 4a). Similarly, the mice vaccinated with S1163 (O9, Vi^+^, ΔP_tviA_::P_ssaG_ Δ*cya* Δ*crp*), S1167 (O9, Vi^+^, ΔP_tviA_::P_ssaG_ Δ*vexE* Δ*crp* Δ*cya*), and S1168 (O9, Vi^+^, ΔP_tviA_::P_ssaG_ Δ*vexA-E* Δ*crp* Δ*cya*) mounted a significantly higher anti-*S*. Enteritidis LPS immune response than the other non-O9 serotype vaccine candidates (Fig. 4b). The IgG2a responses to LPS were significantly higher than the IgG1 responses, indicating that Th1-type cellular immunity was the dominant immune response after immunization (Supplementary Fig. S5). S1114 induced significantly higher levels of anti-*S*. Typhimurium LPS mucosal IgA responses than S1148 or the non-O4 serotype vaccine candidates (Fig. 4c). Similarly, S1163, S1167, and S1168 induced significantly higher levels of anti-*S*. Enteritidis LPS IgA responses than S1114 (Fig. 4d).

Only the two strains producing surface-anchored Vi, S1148 (O4, Vi^+^, ΔP_tviA_::P_ssaG_ Δ*cya* Δ*crp*) and S1163 (O9, Vi^+^, ΔP_tviA_::P_ssaG_ Δ*cya* Δ*crp*), produced a significant anti-Vi serum antibody response (Fig. 4e). This suggests that surface presentation is important for recognition by the immune system. There were no significant differences in the IgG responses against the *S*. Typhimurium OMPs (Fig. 4f). The negative control groups (BSG) in each test mounted no detectable immune responses.

### Serum complement-mediated *S*. Typhi killing is dependent upon anti-Vi and anti-O9 antibodies

**Fig. 5:**
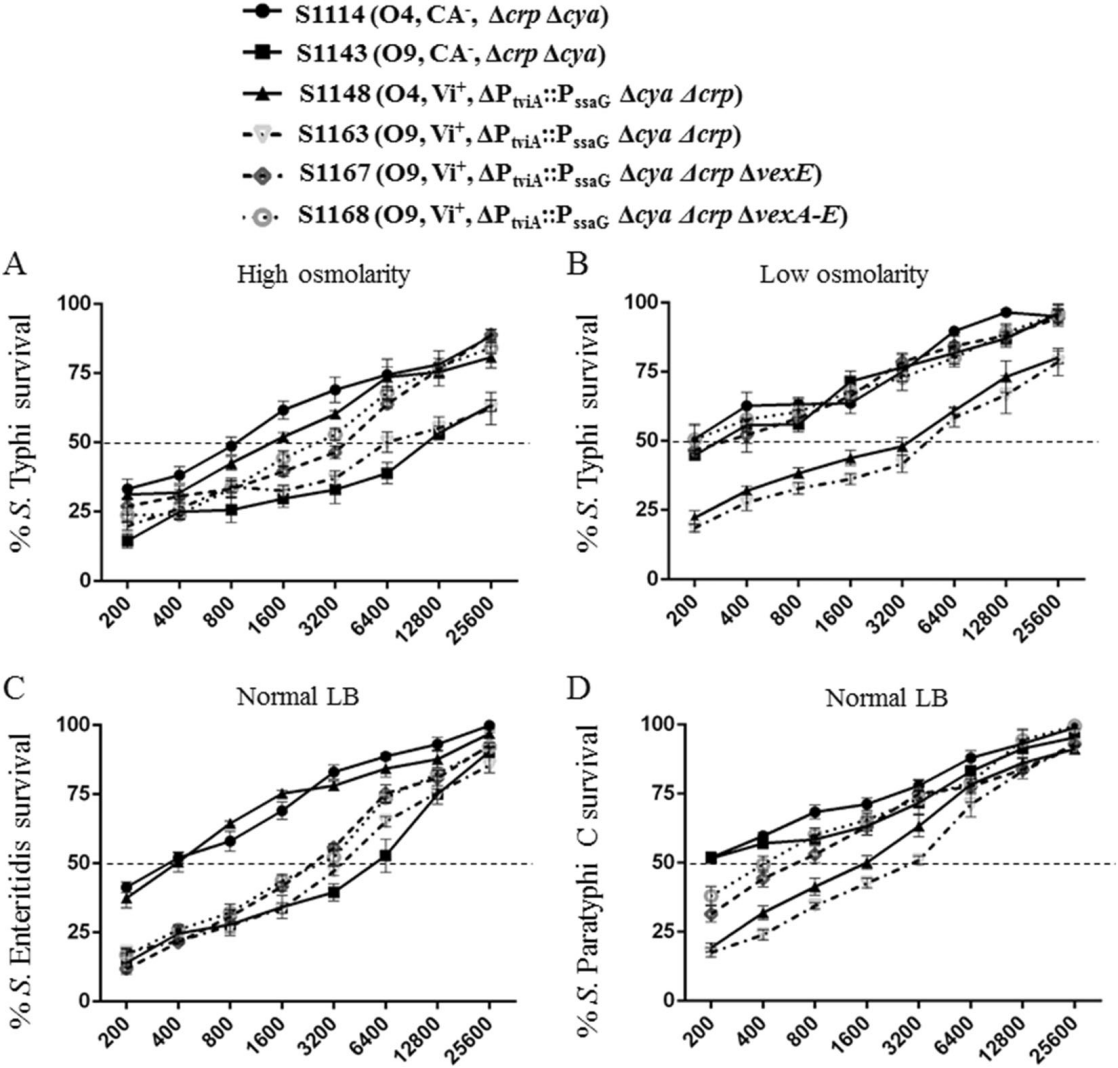
Serum bactericidal activity. Serum bactericidal assays (SBA) were performed with pooled sera from the indicated groups against wild-type *S*. Typhi (**a**, **b**), S. Enteritidis (**c**), and S. Paratyphi C (**d**). Strains were grown in LB containing 500 mM NaCl (**a**), 0 ml NaCl (**b**), or the standard 170 mM NaCl (**c**, **d**). SBA activity at each serum dilution is presented as a percentage of the CFU recovered from the negative control, which contained bacteria and complement only. The error bars represent standard error

To evaluate the functionality of anti-Vi and anti-O-antigen serum antibodies induced by our vaccine strains, we performed the serum bactericidal assays (SBAs) using pooled sera from vaccinated mice. When wild-type *S*. Typhi was grown in high-osmolarity media, conditions expected to reduce Vi production, the serum antibodies induced by both S1143 (O9, CA^−^, Δ*crp* Δ*cya*) and S1163 (O9, Vi^+^, ΔP_tviA_::P_ssaG_ Δ*cya* Δ*crp*) had high levels of SBA activity against *S*. Typhi, with >50% growth inhibition observed at a serum dilution of approximately 1:6400 (Fig. 5a). The serum antibodies induced by S1167 (O9, Vi^+^, ΔP_tviA_::P_ssaG_ Δ*vexE* Δ*crp* Δ*cya*) and S1168 (O9, Vi^+^, ΔP_tviA_::P_ssaG_ Δ*vexA-E* Δ*crp* Δ*cya*) exhibited the next highest levels of SBA activity, with 50% growth inhibition of *S*. Typhi observed in serum diluted approximately 1:3200. The bacteriocidal titers induced by S1114 (O4, CA^−^, Δ*crp* Δ*cya*) and S1148 (O4, Vi^+^, Δ*cya* Δ*crp*) were the lowest, with >50% growth inhibition of *S*. Typhi observed at serum dilutions of 1:800 to 1:1600, respectively (Fig. 5a). After growth in low-osmolarity media, conditions which induce Vi antigen production, *S*. Typhi was no longer susceptible to killing by sera from mice immunized with strains S1114, S1143, S1167, and S1168, suggesting that Vi was effectively masking the outer surface of the cells. However, sera from mice immunized with S1163 (O9, Vi^+^, ΔP_tviA_::P_ssaG_ Δ*cya* Δ*crp*) or S1148 (O4, Vi^+^, ΔP_tviA_::P_ssaG_ Δ*cya* Δ*crp*) exhibited high titers of SBA activity against *S*. Typhi, with >50% growth inhibition observed at a 1:3200 dilution (Fig. 5b). We also evaluated the SBA activity against wild-type *S*. Enteritidis (O9, Vi^-^) and *S*. Paratyphi C (O6, O7, Vi^+^). The serum antibodies induced by O9^+^ strains S1143, S1163, S1167, and S1168 exhibited the highest SBA activity against *S*. Enteritidis, with >50% growth inhibition observed at a dilution of approximately 1:3200 (Fig. 5c). The serum antibodies induced by Vi^+^ strains S1148 and S1163 exhibited high SBA activity against *S*. Paratyphi C, with >50% growth inhibition at dilutions of approximately 1:1600 and 1:3200, respectively. These results indicate that the anti-O9 and anti-Vi serum antibodies elicited by our live attenuated vaccines have relevant biological activity.

### Protective efficacy of live attenuated vaccines against challenge with wild-type *S*. Typhimurium and *S*. Enteritidis

We investigated whether our live vaccines would stimulate protective immunity against wild-type *S*. Typhimurium and *S*. Enteritidis challenge.^37^ Since *S*. Typhi does not effectively colonize nor cause disease in mice, we decided to challenge with a mouse-virulent strain of *S*. Enteritidis. Note that the vaccines converted to serotype O9 induced high levels of anti-O9 antibody immune responses with effective SBA activity against both *S*. Enteritidis and *S*. Typhi, which share the same O9 O-antigen polysaccharide. We also challenged a separate group of mice with *S*. Typhimurium to evaluate the ability of our strains to cross-protect.

All the mice survived the 100× LD_50_ challenge with *S*. Typhimurium, indicating that all of our strains provide a high level of homologous protection, even when the strains were converted to express the O9 epitope (Supplementary Fig. S6A). Mice vaccinated with S1143 (O9, CA^−^, Δ*crp* Δ*cya*) and S1163 (O9, Vi^+^, ΔP_tviA_::P_ssaG_ Δ*cya* Δ*crp*) were completely protected against an oral challenge of 100× LD_50_ of wild-type *S*. Enteritidis. Eighty percent of the mice in groups vaccinated with strains S1163 (O9, Vi^+^, ΔP_tviA_::P_ssaG_ Δ*cya* Δ*crp*), S1167 (O9, Vi^+^, ΔP_tviA_::P_ssaG_ Δ*vexE* Δ*crp* Δ*cya*), and S1168 (O9, Vi^+^, ΔP_tviA_::P_ssaG_ Δ*vexA-E* Δ*crp* Δ*cya*) survived challenge, indicating a high level of protection. Mice vaccinated with S1114 (O4, CA^−^, Δ*crp* Δ*cya*) and S1148 (O4, Vi^+^, ΔP_tviA_::P_ssaG_ Δ*cya* Δ*crp*) were not protective, although there was a slight delay in time to death (Supplementary Fig. S6B).

## Discussion

The first licensed live vaccine for preventing *S*. Typhi infection was *S*. Typhi Ty21a, which was developed to mimic a natural mucosal infection. While this vaccine is well tolerated, it has several drawbacks, including an unclear understanding of the mutations responsible for its attenuation and the requirement of multiple doses for optimal immunogenicity. With the goal of developing live vaccines requiring only a single oral dose, multiple research groups have engineered new live attenuated vaccines to induce serum anti-Vi antibody responses. A series of potential live vaccine candidates (CVD908, CVD908-*htrA*, CVD909, Ty800, and ZH9) were designed and developed.^7,8,38^ However, none of them stimulate high levels of anti-Vi serum antibodies,^7,8,38^ although CVD909 has shown promise when used as the priming dose prior to boosting with purified Vi.^7^

The production of Vi capsule is highly regulated in vivo. In the human intestinal lumen, a high-osmolarity environment, *S*. Typhi displays a non-capsulated, flagellated, and invasive phenotype associated with a functional type III secretion system 1 (T3SS-1). Transition through the intestinal epithelium rapidly induces the *tviA* gene, which upregulates Vi capsule production to protect against complement deposition and phagocytosis while simultaneously masking LPS, a TLR4 agonist, and repressing the biosynthesis of flagella and T3SS-1 effectors.^20,21^ Shortly after invasion of the gut epithelium, *S*. Typhi encounters macrophages and other phagocytic cells in gut-associated lymphoid tissues. The interaction between *S*. Typhi and macrophages results in downregulation of Vi capsular expression,^35^ which is believed to enable evasion of anti-Vi immune responses, and upregulation of type III secretion system 2 biosynthesis, which is essential for *S*. Typhi survival in macrophages.^39^ Because anti-group D LPS antibodies can enhance the protection provided by anti-Vi serum antibodies,^11^ we designed a *S*. Typhimurium vaccine to synthesize both Vi capsule and O9 O-antigen to elicit robust antibody responses against both polysaccharides (Fig. 1 and Supplementary Fig. S1). When *S*. Typhi is transiting through the intestinal epithelium, the exposed O-antigen polysaccharides on the surface are vulnerable to be targeted by anti-O-antigen antibodies. Once *S*. Typhi invades in intestinal epithelium, the upregulated expression of Vi capsular would be attacked by anti-Vi antibodies.

Janis et al. were the first to generate a live attenuated *S*. Typhimurium vaccine with in vivo-inducible Vi expression that stimulated high anti-Vi antibodies in the serum after single oral administration.^10^ In their report, Hfr conjugation between *S*. Typhi Ty2 and *S*. Typhimurium C5 was used to generate a Vi-positive *S*. Typhimurium (C5.507 Vi^+^). In this study, we replaced the entire CA operon with a functional *viaB* locus and with no extraneous *S*. Typhi sequences, resulting in *S*. Typhimurium mutant S1123 (O4, Vi^+^,) (Fig. 1a). CA is a surface exopolysaccharide produced by many enteric bacteria, and deletion of the entire CA operon has no influence on virulence but increases the synthesis and exposure of heterologous antigens in *Salmonella*.^40^ Additionally, the original O4 serotype in *S*. Typhimurium was converted to O9 by replacing the allelic *abe* gene with *prt*-*tyv*_D1_ from *S*. Enteritidis (Fig. 1e). While *S*. Typhi and *S*. Enteritidis share the same O9 O-antigen epitope, their O-antigens differ in polymer length (Fig. 2a). *S*. Enteritidis possess a long O-antigen polysaccharide while a short O9 O-antigen polysaccharide has evolved in *S*. Typhi due to inactivation of the *fepE* gene, a regulator of long O-antigen polysaccharide synthesis.^32,41^ The Vi capsular instead plays this protective role on the surface by covering the short O9 O-antigen polysaccharide in *S*. Typhi.^32^

In vitro analyses indicated that all Vi^+^ strains remained P22 transducible, indicating that Vi production did not block P22 binding under the conditions of the assay (Supplementary Table S2). This is consistent with our expectation that the P_ssaG_ promoter is not active during growth in Luria-Bertani (LB). Swimming motility was unaffected in all strains except for S1160 (O9, Vi^+^, ΔP_tviA_::P_ssaG_ Δ*vexA-E*), which exhibited a slight defect, indicating that intracellular accumulation of Vi perturbed flagellar gene regulation, flagellar protein transport and assembly, or both.

Vi-producing strains S1137, S1151, S1159, and S1160 were attenuated approximately 1000-fold (Supplementary Table S2). This decrease in virulence is likely to be related to in vivo synthesis of the heterologous Vi antigen. The attenuated virulence did not impact the immune responses induced by the S1148 (O4, Vi^+^, ΔP_tviA_::P_ssaG_ Δ*cya* Δ*crp*) and S1163 (O9, Vi^+^, ΔP_tviA_::P_ssaG_ Δ*cya* Δ*crp*) vaccine strains, which produce surface-exposed Vi. However, immunization with strains S1167 (O9, Vi^+^, ΔP_tviA_::P_ssaG_ Δ*vexE* Δ*crp* Δ*cya*) and S1168 (O9, Vi^+^, ΔP_tviA_::P_ssaG_ Δ*vexA-E* Δ*crp* Δ*cya*) did not result in anti-Vi IgG in the serum (Fig. 4f), indicating that only surface exposed, lipid-anchored^24^ Vi is immunogenic when delivered by live *Salmonella*. The fact that S1163 (O9, Vi^+^, ΔP_tviA_::P_ssaG_ Δ*cya* Δ*crp*) could induce strong anti-O9 serum IgG responses was encouraging (Fig. 4d), demonstrating that our vaccine design was adequate to allow presentation of both antigens. One explanation for inducing antibodies against both saccharides is that the O-antigens produced by our construct are comparable to the length of Vi capsular and are longer than native *S*. Typhi O-antigen.

The primary functions of anti-Vi and anti-O-antigen antibodies are to direct complement deposition on the bacterial cell surface and to promote antibody-mediated phagocytosis by macrophages.^11^ While opsonization with anti-Vi antibodies is effective, they would not be expected to impact *S*. Typhi cells in an environment where Vi production is downregulated. Conversely, anti-O9 antibodies are not effective in the presence of Vi^11^ (Fig. 5). Thus we postulated that the synergistic actions of both anti-Vi and anti-O9 antibodies may overcome this innate immune evasion mechanism. We used the SBA assay to investigate this idea (Fig. 5). Indeed, when grown at high osmolarity (Vi-suppressing conditions), *S*. Typhi was sensitive to mouse sera containing anti-O9 antibodies and no anti-Vi antibodies (O9^+^, Vi^−^) (Fig. 5a, S1143 group), but was not sensitive to Vi^+^, O9^−^ mouse sera (group S1148). Conversely, *S*. Typhi cells grown at low osmolarity (Vi-inducing conditions) was sensitive to Vi^+^, O9^−^ mouse sera (Fig. 5b, S1148 group) but was not sensitive to Vi^−^, O9^+^ sera (S1143 group). The functionality of the anti-O9 and anti Vi-antibodies was confirmed by their ability to direct complement-mediated killing of *S*. Enteritidis (O9, Vi^−^) and S. Paratyphi C (O7, Vi^+^), respectively (Fig. 5c, d). Importantly, the sera from mice immunized with strain S1163 (O9^+^, Vi^+^) contained both anti-O9 and anti-Vi antibodies (Fig. 4b, e) and directed efficient complement-mediated killing of *S*. Typhi cells grown at either low- or high-osmolarity (Fig. 5a, b). This indicates that the strategy used to construct strain S1163 resulted in antibodies capable of attacking *S*. Typhi regardless of its Vi status. The protective efficacy of the anti-O9 response was further supported by our challenge results showing that immunization with S1163 resulted in complete protection against *S*. Enteritidis (Supplementary Fig. S4B).

Most live typhoid vaccine strains (e.g., *S*. Ty21a,^42^ Ty800,^13^ M01ZH09,^43^ CVD908^44^) and *Salmonella* strains designed to deliver heterologous antigens to combat human disease, such as χ9633 and χ9640,^45^ were derived from *S*. Typhi, which is human host-restricted and able to colonize humans systemically, including tissues such as liver, spleen, and bone marrow. Since wild-type *S*. Typhimurium does not penetrate to the deeper tissues, typically invading only local intestinal epithelial tissue, attenuated *S*. Typhimurium strains are often not considered for human use. However, there have been two clinical trials in which they were evaluated. Human subjects orally immunized with attenuated *S*. Typhimurium strains LH1160^46^ and WT05^47^ developed strong mucosal or serological responses. Most *S*. Typhi vaccines carry deletion mutations associated with systemic dissemination, such as *phoPQ*, *htrA*, and *ssaV*, with no bacteremia observed during vaccination, indicating that the protection would theoretically depend largely on local immune induction sites.^9^ Therefore, we propose that a properly attenuated *S*. Typhimurium strain could also be used as a live typhoid vaccine and as a vaccine vector for human use.

**Fig. 6:**
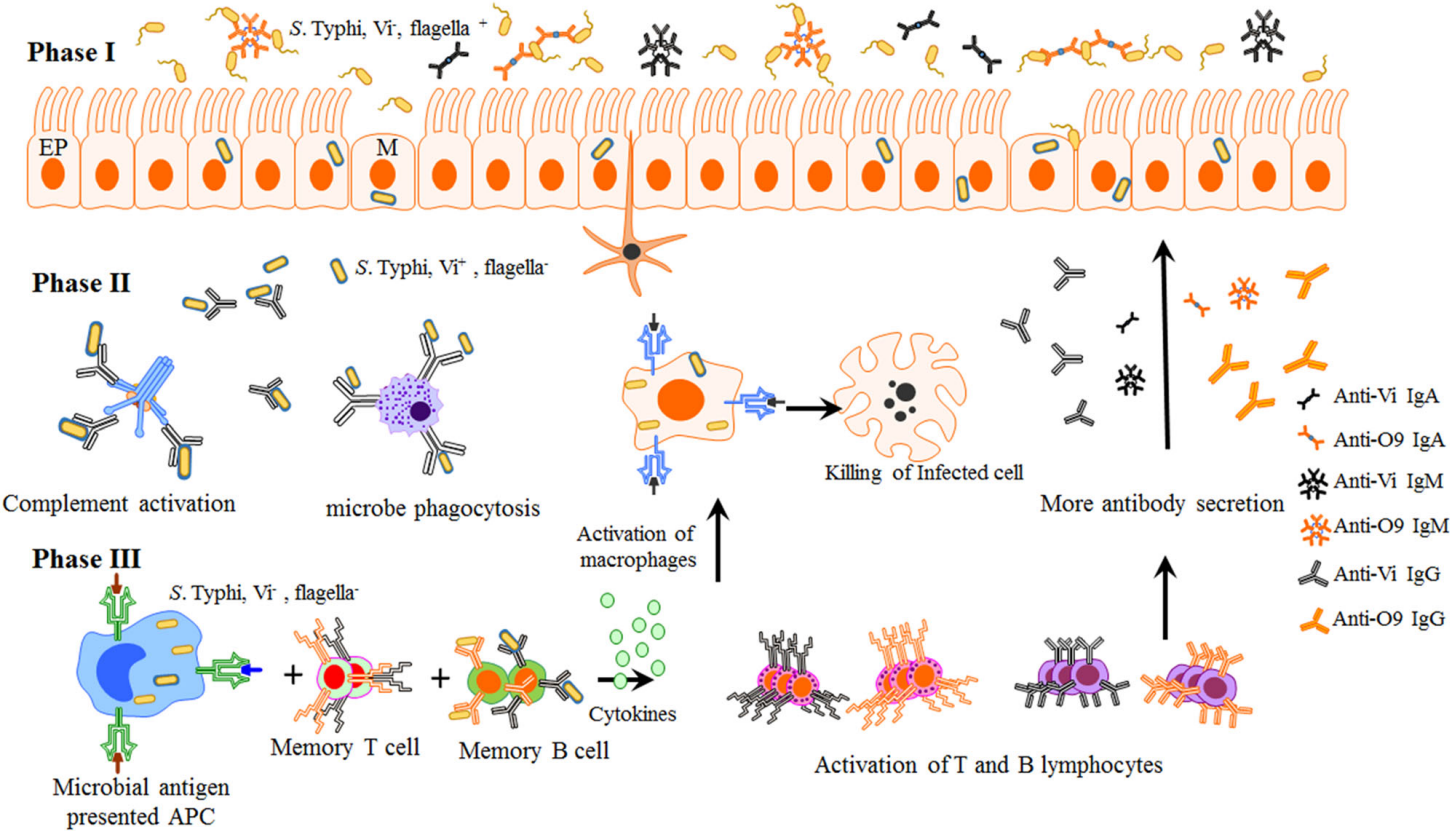
A model summarizing the various levels of immunity induced by *S*. Typhimurium vaccine strain S1163 to inhibit *S*. Typhi infection. Phase I. Anti-O9 mucosal antibodies inhibit *S*. Typhi attachment and invasion of the gut epithelium. Phase II. Anti-Vi antibodies support complement deposition of *S*. Typhi and facilitate uptake of *S*. Typhi by phagocytic cells. Phase III. Induction of memory T and B cells and activation of T and B lymphocytes producing anti-O9 and anti-Vi antibodies. EP intestinal epithelium, M microfold cells

We envision that the immune responses elicited by orally administered *S*. Typhimurium vaccine S1163 (O9, Vi^+^, ΔP_tviA_::P_ssaG_ Δ*cya* Δ*crp*) will provide protection as follows. When *S*. Typhi arrives at intestinal mucosal surfaces, it displays a non-capsulated, flagellated, and invasive phenotype susceptible to secreted anti-O9 IgA and IgM (Fig. 6, Phase I). *S*. Typhi cells that survive this first line of defense transit through the intestinal barrier. Vi capsule polysaccharides are rapidly produced and the flagellar synthesis is repressed. Vi-encapsulated *S*. Typhi will then encounter macrophages, dendritic cells, neutrophils, and complement systems. At this stage, vaccine-induced anti-Vi antibody (IgG) will enhance the bactericidal activities of these host systems (Phase II). Some *S*. Typhi cells will be captured by antigen-presenting cells (macrophages or dendritic cells) to interact with memory T cells. Vi capsule is shed within macrophages, leaving *S*. Typhi cells uncapsulated, allowing efficient processing to facilitate activation of B cells and T cells. More and efficient anti-Vi and anti-O9 antibodies (IgA, IgG) produced by activated B cell and activated T cell (cytotoxic T cells or natural killing cells) will aid in eliminating the infected cells and clearing systemic infection (Phase III).

Our work highlights the possibility of developing live attenuated *S*. Typhimurium vaccines to prevent typhoid fever by targeting the protective Vi capsular and O9 O-polysaccharide antigens simultaneously. This strategy was also effective at providing strong protection against *S*. Typhimurium challenge (Supplementary Fig. S4). This work provides a mechanism for developing multi-valent live attenuated vaccine against group B and group D *Salmonella* infections.

## Materials and methods

Additional materials and methods could be found in Supplementary Text S1.

### Bacteria, plasmids, and culture conditions

Bacteria and plasmids used in this study are listed in Table 1. *Escherichia coli* and *Salmonella* strains were aerobically grown at 37 °C in LB broth or on LB agar. To induce maximum Vi capsular production, wild-type *S*. Typhi was grown aerobically in low-osmolarity broth (10 g/l tryptone, 5 g/l yeast extract, with no added NaCl) to mid-log phase at 37 °C.^17^ To inhibit Vi capsular synthesis, wild-type *S*. Typhi were grown in high-osmolarity LB broth (approximately 500 mM NaCl). When necessary, chloramphenicol was added at 25 µg/ml for selection of transconjugants.

**Table 1:** Bacterial strains and plasmids used in this study

Strains or plasmids	Description	Source
*S*. Typhimurium		
S1032	Δ(*wza*-*wcaM*)-1 Δ*abe*-1::*prt*-*tyv*_D1_	This study
S1114	Δ(*wza*-*wcaM*)-1 Δ*crp*-24 Δ*cya*-25	This study
S1143	Δ(*wza*-*wcaM*)-1 Δ*abe*-1::*prt*-*tyv*_D1_ Δ*crp*-24 Δ*cya*-25	This study
S1123	Δ(*wza*-*wcaM*)-1::*viaB*	This study
S1130	Δ(*wza*-*wcaM*)-1::*viaB* Δ*abe*-1::*prt*-*tyv*_D1_	This study
S1137	Δ(*wza*-*wcaM*)1::*viaB* ΔP_tviA1_::P_ssaG1_	This study
S1151	Δ(*wza*-*wcaM*)-1::*viaB* Δ*abe*-1::*prt*-*tyv*_D1_ ΔP_tviA1_::P_ssaG1_	This study
S1159	Δ(*wza*-*wcaM*)-1::*viaB* Δ*abe*::*prt*-*tyv*_D1_ ΔP_tviA1_::P_ssaG1_ Δ*vexE*1	This study
S1160	Δ(*wza*-*wcaM*)-1::*viaB* Δ*abe*-1::*prt*-*tyv*_D1_ ΔP_tviA1_::P_ssaG1_ Δ(*vexA*-*vexE*)-2	This study
S1148	Δ(*wza*-*wcaM*)-1::*viaB* ΔP_tviA1_::P_ssaG1_ Δ*cya*-24 Δ*crp*-25	This study
S1163	Δ(*wza*-*wcaM*)-1::*viaB* Δ*abe*::*prt*-*tyv*_D1_ ΔP_tviA1_::P_ssaG1_ Δ*cya*-24 Δ*crp*-25	This study
S1167	Δ(*wza*-*wcaM*)-1::*viaB* Δ*abe*::*prt*-*tyv*_D1_ ΔP_tviA1_::P_ssaG1_ Δ*vexE*1 Δ*crp*-24 Δ*cya*-25	This study
S1168	Δ(*wza*-*wcaM*)-1::*viaB* Δ*abe*-1::*prt*-*tyv*_D1_ ΔP_tviA1_::P_ssaG1_ Δ(*vexA*-*vexE*)-2 Δ*crp*-24 Δ*cya*-25	This study
Other Salmonella serovars and *E. coli* strains		
S100	*S*. Typhimurium	[36]
S246	*S*. Enteritidis	[36]
S229	*S*. Typhi	CDC
S273	*S*. Paratyphi C	CDC
χ7232	*E. coli endA1 hsdR17* (r_K_^−^, m_k_^+^) *glnV44 thi-1 recA1 gyrA relA1 ∆*(*lacZYA-argF*)*U169 λpir deoR* (ϕ*80dlac* ∆(*lacZ*)*M15*)	[39]
χ7213	*E. coli thi-1 thr-1 leuB6 glnV44 fhuA21 lacY1 recA1 RP4-2-Tc*::Mu λ*pir* ∆*asdA4* ∆*zhf-2*::Tn*10*	[39]
Suicide plasmids		
pYA4278	*sacB* mobRP4 R6K *ori* Cm^+^	[39]
pYA4518	p15a *ori* GFP^+^ Cm^+^	[39]
pSS022	Δ*crp*-24 construction	[30]
pSS023	Δ*cya*-25 construction	[30]
pSS908	Δ*abe*-1 construction	This study
pSS916	Δ*abe*-1::*prt*-*tyv*_D1_ construction	This study
pSS997	Δ(*wza*-*wcaM*)1 construction	This study
pSS1004	Δ(*wza*-*wcaM*)1::*viaB* construction	This study
pSS916	ΔP_tviA1_::P_ssaG1_ construction	This study
pSS1001	Δ*vexE*1 construction	This study
pSS1025	Δ(*vexA*-*vexE*)2 construction	This study

*CDC* Chinese center for disease control and prevention

### Molecular and genetic procedures

Suicide vectors and primers used in this study are listed in Table 1 and Supplemental Table S1, respectively. DNA fragments were assembled using Gibson Assembly Master Mix according to the manufacturer’s instructions (New England BioLabs). *sacB* gene-based sucrose counter-selectable suicide vectors were used to construct unmarked deletion and/or insertion mutations in *S*. Typhimurium.^48^ Specifically, for deletion mutations, two homologous DNA fragments, the upstream and downstream regions of the gene or operon being deleted, were amplified. After purification, these two fragments were fused by PCR and cloned into the pYA4278 suicide vector. The conjugational transfer of recombinant suicide vectors to *S*. Typhimurium was performed using the suicide vector donor strain χ7213. Transconjugants were selected on chloramphenicol agar without supplemental DAP. The second homologous recombination event, resulting in excision of the suicide vector from a *S*. Typhimurium chromosome, was selected on 10% sucrose LB plates without sodium chloride and grown at 30 °C. Successful gene deletion mutations were confirmed by PCR screening and DNA sequencing. For the insertion mutations, the genes or operon being inserted and the suicide vector backbone containing the directed insertion site were amplified. After purification, these two linear DNA fragments were assembled sequentially, resulting in a new circular suicide vector with new genes or operons replacing those previously deleted. The subsequent insertion mutation processes were the same as those described above for the deletion mutations. The deletion and insertion mutations constructed for this study are illustrated in Fig. 1.

### LPS silver staining and western blotting

LPS silver staining were prepared, separated, and visualized using the method provided by Hitchcock and Brown.^49^ For western blotting, anti-O-antigen single-factor rabbit antisera (BD Biosciences) or anti-Vi polymer rabbit antisera (BD Biosciences) were used to probe the blots in polyvinylidene difluoride membranes. Then the membranes were incubated with anti-rabbit horseradish peroxidase-conjugated antibodies (Sigma, St. Louis, MO, USA). Patterns were detected by chemiluminescence using western ECL blotting Substrates (Bio-Rad, Hercules, CA, USA).

### In vitro Vi capsular expression analysis via immunostaining

The RAW264.7 macrophage cells were seeded onto glass coverslips in 12-well plates prior to infection. The bacteria were added to each well at a multiplicity of infection of 10:1 and incubated for 1 h. After infection, the plates were washed three times with phosphate-buffered saline (PBS) and fixed with 4% paraformaldehyde for 15 min. Cells were permeabilized for 10 min in 0.1% Triton X-100, blocked with 5% bovine serum albumin for 1 h, and incubated with a rabbit anti-Vi polyclonal antibody (1:100 dilution) (BD Biosciences) for 16 h at 4 °C. Subsequently, the plates were washed three times with PBS and then incubated with an Alexa Fluor 568-conjugated goat anti-rabbit antibody (1:200 dilution) (Life Technologies) at room temperature for 1 h. Finally, the cells were treated with 4’, 6-diamidino-2-phenylindole (Invitrogen) for 15 min. All the coverslips were washed with PBS three times and mounted onto glass slides. Fluorescence signals were examined under a microscope (Eclipse 80i, Nikon, Japan) at 10 × 40 magnification, and cell images were captured with a Spot camera using the Spot software (Diagnostic Instruments, Sterling Heights, MI, USA).

### Vaccination and immune response measurement

Animal studies were conducted in compliance with the regulations stated in the Guide for the Care and Use of Laboratory Animals, which was approved by Sichuan Agricultural University Institutional Animal Care and Use Committee (Ya’an, China; Approval No. 2011028).

Twenty-four 6-week-old female BALB/c mice per group were vaccinated orally on day 0 with 20 μl BSG containing approximately 1 × 10^9^ CFU vaccine strains and boosted on day 28 with the same dose. Blood samples and vaginal secretions were collected from randomly selected 12 mice in each group on day 56 after the booster immunization, and the mice in each group were randomly challenged orally on day 63 with 5 × 10^7^ CFU of *S*. Typhimurium or *S*. Enteritidis (~100 times the LD_50_).^37^ *S*. Typhimurium and *S*. Enteritidis LPS (Sigma, St. Louis, MO, USA) were used to measure immune responses. IgG and IgA antibodies specific to *S*. Typhimurium and *S*. Enteritidis LPS in the serum or vaginal secretions were measured using the quantitative enzyme-linked immunosorbent assay (ELISA) as described previously.^50^ Antibody concentrations were calculated based on absorbance values and the standard curve. Quantitative measurement of the mouse IgG antibody against the *S*. Typhi Vi polysaccharide in the serum was achieved using the Typhoid Vi IgG ELISA Kit (cat. #990-520-MTG, Alpha Diagnostic Intl. Inc. San Antonio, TX, USA) according to the manufacturer’s instructions. IgG concentrations (U/ml) specific to Vi were calculated based on calibrator value graphs.

### Serum bactericidal activity assay

SBA was performed as previously described with a few modifications,^51^ *S*. Typhi, *S*. Paratyphi C, and *S*. Enteritidis were grown to an OD_600_ of 0.6 in either low- (0 ml NaCl), normal- (170 mM NaCl) or high-osmolarity (500 mM NaCl) LB broth as indicated. After centrifugation and resuspension, the log-phase cultures were diluted in PBS to a concentration of approximately 1 × 10^4^ CFU/ml. Pooled vaccinated and non-vaccinated mouse sera were heat-inactivated at 56 °C for 20 min, and two-fold serial dilutions in PBS starting from 1:200 to 1:25,600 were made in 96-well plates. Optimal SBA results were achieved by combining 25 µl of active baby rabbit complement (25% final concentration) with 15 µl of PBS, 50 µl of diluted mice pooled sera, and 10 µl of diluted bacteria (~350 CFU). In total, 10 µl of the mixture from each well was spread on LB agar plates after 60 min to assess the bactericidal activity. The spread LB agar plates were incubated overnight at 37 °C, and the viable CFUs were counted the next day. The negative control contained only bacteria and complement, and the bactericidal activity was determined as the percentage of CFU counts in each pooled sera dilution compared to the CFU counts of the negative control. The SBA graphs depict the percentage of bacterial growth as a function of the sera antibodies in each diluted pool.

### Statistical analysis

Data were analyzed using the GraphPad Prism 5 software (Graph Software, San Diego, CA) by one-way or two-way analysis of variance followed by Tukey’s multiple-comparison post-test. Kaplan–Meier survival curve comparisons were calculated by comparing two groups at each time point through the log-rank (Mantel–Cox) test. The data were expressed as the means ± SEM. *P* < 0.05 was considered statistically significant.

### Data availability statement

The data that support the findings of this study are available from the corresponding author upon reasonable request.

## Electronic supplementary material

Text S1

Table S1

Table S2

Supplementary figures

## References

[1] House D, Bishop A, Parry C, Dougan G, Wain J (2001). Typhoid fever: pathogenesis and disease. Curr. Opin. Infect. Dis..

[2] Crump JA, Luby SP, Mintz ED (2004). The global burden of typhoid fever. Bull. World Health Organ..

[3] Bhan MK, Bahl R, Bhatnagar S (2005). Typhoid and paratyphoid fever. Lancet.

[4] Zaki SA, Karande S (2011). Multidrug-resistant typhoid fever: a review. J. Infect. Dev. Ctries..

[5] Khan MI (2012). Effectiveness of Vi capsular polysaccharide typhoid vaccine among children: a cluster randomized trial in Karachi, Pakistan. Vaccine.

[6] Owais A, Sultana S, Zaman U, Rizvi A, Zaidi AK (2010). Incidence of typhoid bacteremia in infants and young children in southern coastal Pakistan. Pediatr. Infect. Dis. J..

[7] Wahid R, Salerno-Goncalves R, Tacket CO, Levine MM, Sztein MB (2007). Cell-mediated immune responses in humans after immunization with one or two doses of oral live attenuated typhoid vaccine CVD 909. Vaccine.

[8] Tacket CO, Pasetti MF, Sztein MB, Livio S, Levine MM (2004). Immune responses to an oral typhoid vaccine strain that is modified to constitutively express Vi capsular polysaccharide. J. Infect. Dis..

[9] Galen, J. E., Buskirk, A. D., Tennant, S. M. & Pasetti, M. F. Live attenuated human *Salmonella* vaccine candidates: tracking the pathogen in natural infection and stimulation of host immunity. *EcoSal Plus* **7**, 1–17 (2016). 10.1128/ecosalplus.esp-0010-2016 PMC5119766 27809955

[10] Janis C (2011). In vivo regulation of the Vi antigen in *Salmonella* and induction of immune responses with an in vivo-inducible promoter. Infect. Immun..

[11] Hart PJ (2016). Differential killing of *Salmonella enterica* serovar Typhi by antibodies targeting Vi and lipopolysaccharide O:9 antigen. PLoS ONE.

[12] Levine MM, Tacket CO, Sztein MB (2001). Host–*Salmonella* interaction: human trials. Microbes Infect..

[13] Hohmann EL, Oletta CA, Killeen KP, Miller SI (1996). *phoP*/*phoQ*-deleted *Salmonella* typhi (TY800) is a safe and immunogenic single dose typhoid fever vaccine in volunteers. J. Infect. Dis..

[14] Heyns K, Kiessling G (1967). Strukturaufklärung des vi-antigens aus *Citrobacter freundii* (*E. coli*) 5396/38. Carbohyd. Res..

[15] Wilson RP (2011). The Vi capsular polysaccharide prevents complement receptor 3-mediated clearance of *Salmonella enterica* serotype Typhi. Infect. Immun..

[16] Pickard D (1994). Characterization of defined *ompR* mutants of *Salmonella typhi*: *ompR* is involved in the regulation of Vi polysaccharide expression. Infect. Immun..

[17] Arricau N (1998). The RcsB–RcsC regulatory system of *Salmonella typhi* differentially modulates the expression of invasion proteins, flagellin and Vi antigen in response to osmolarity. Mol. Microbiol..

[18] Santander J, Wanda SY, Nickerson CA, Curtiss R (2007). Role of RpoS in fine-tuning the synthesis of Vi capsular polysaccharide in *Salmonella enterica* serotype Typhi. Infect. Immun..

[19] Wetter M (2012). Molecular characterization of the *viaB* locus encoding the biosynthetic machinery for Vi capsule formation in *Salmonella* Typhi. PLoS ONE.

[20] Winter SE, Raffatellu M, Wilson RP, Russmann H, Baumler AJ (2008). The *Salmonella enterica* serotype Typhi regulator TviA reduces interleukin-8 production in intestinal epithelial cells by repressing flagellin secretion. Cell. Microbiol..

[21] Winter SE (2014). *Salmonella enterica* Serovar Typhi conceals the invasion-associated type three secretion system from the innate immune system by gene regulation. PLoS Pathog..

[22] Winter SE (2009). The TviA auxiliary protein renders the *Salmonella enterica* serotype Typhi RcsB regulon responsive to changes in osmolarity. Mol. Microbiol..

[23] Winter SE (2010). A rapid change in virulence gene expression during the transition from the intestinal lumen into tissue promotes systemic dissemination of *Salmonella*. PLoS Pathog..

[24] Liston SD, Ovchinnikova OG, Whitfield C (2016). Unique lipid anchor attaches Vi antigen capsule to the surface of *Salmonella enterica* serovar Typhi. Proc. Natl. Acad. Sci. USA.

[25] Jörbeck HJ, Svenson SB, Lindberg AA (1979). Immunochemistry of *Salmonella* O-antigens: specificity of rabbit antibodies against the O-antigen 4 determinant elicited by whole bacteria and O-antigen 4 specific saccharide-protein conjugates. J. Immunol..

[26] Reeves PR, Cunneen MM, Liu B, Wang L (2013). Genetics and evolution of the *Salmonella* galactose-initiated set of O antigens. PLoS. ONE.

[27] Samuel G, Reeves P (2003). Biosynthesis of O-antigens: genes and pathways involved in nucleotide sugar precursor synthesis and O-antigen assembly. Carbohyd. Res..

[28] Li P, Kong Q (2017). Front. Immunol..

[29] McKelvie ND (2004). Expression of heterologous antigens in *Salmonella* Typhimurium vaccine vectors using the in vivo-inducible, SPI-2 promoter, *ssaG*. Vaccine.

[30] Curtiss R, Kelly SM (1987). *Salmonella typhimurium* deletion mutants lacking adenylate cyclase and cyclic AMP receptor protein are avirulent and immunogenic. Infect. Immun..

[31] Liu X (2016). Attenuated *Salmonella* Typhimurium delivery of a novel DNA vaccine induces immune responses and provides protection against duck enteritis virus. Vet. Microbiol..

[32] Crawford RW (2013). Loss of very-long O-antigen chains optimizes capsule-mediated immune evasion by *Salmonella enterica* serovar Typhi. mBio.

[33] Wilson RP (2008). The Vi-capsule prevents Toll-like receptor 4 recognition of *Salmonella*. Cell. Microbiol..

[34] Tran QT (2009). The *Salmonella enterica* serotype Typhi Vi capsular antigen is expressed after the bacterium enters the ileal mucosa. Infect. Immun..

[35] Faucher SP, Porwollik S, Dozois CM, McClelland M, Daigle F (2006). Transcriptome of *Salmonella enterica* serovar Typhi within macrophages revealed through the selective capture of transcribed sequences. Proc. Natl. Acad. Sci. USA.

[36] Valdivia RH, Falkow S (1997). Fluorescence-based isolation of bacterial genes expressed within host cells. Science.

[37] Liu Q (2016). Immunogenicity and cross-protective efficacy induced by outer membrane proteins from *Salmonella* Typhimurium mutants with truncated LPS in mice. Int. J. Mol. Sci..

[38] Tacket CO, Levine MM (2007). CVD 908, CVD 908-htrA, and CVD 909 live oral typhoid vaccines: a logical progression. Clin. Infect. Dis..

[39] Kuhle V, Hensel M (2004). Cellular microbiology of intracellular *Salmonella enterica*: functions of the type III secretion system encoded by *Salmonella* pathogenicity island 2. Cell. Mol. Life Sci..

[40] Li P (2017). Reversible synthesis of colanic acid and O-antigen polysaccharides in *Salmonella* Typhimurium enhances induction of cross-immune responses and provides protection against heterologous *Salmonella* challenge. Vaccine.

[41] Murray GL, Attridge SR, Morona R (2003). Regulation of *Salmonella typhimurium* lipopolysaccharide O antigen chain length is required for virulence; identification of FepE as a second Wzz. Mol. Microbiol..

[42] Kopecko DJ (2009). Genetic stability of vaccine strain *Salmonella* Typhi Ty21a over 25 years. Int. J. Med. Microbiol..

[43] Kirkpatrick BD (2006). Evaluation of *Salmonella enterica* serovar Typhi (Ty2 aroC-ssaV-) M01ZH09, with a defined mutation in the *Salmonella* pathogenicity island 2, as a live, oral typhoid vaccine in human volunteers. Vaccine.

[44] Tacket CO (1997). Safety of live oral *Salmonella typhi* vaccine strains with deletions in htrA and aroC aroD and immune response in humans. Infect. Immun..

[45] Frey SE (2013). A phase I, dose-escalation trial in adults of three recombinant attenuated *Salmonella* Typhi vaccine vectors producing *Streptococcus pneumoniae* surface protein antigen PspA. Vaccine.

[46] Angelakopoulos H, Hohmann EL (2000). Pilot study of *phoP*/*phoQ*-deleted *Salmonella enterica* serovar Typhimurium expressing *Helicobacter pylori* urease in adult volunteers. Infect. Immun..

[47] Hindle Z (2002). Characterization of *Salmonella enterica* derivatives harboring defined *aroC* and *Salmonella* pathogenicity island 2 type III secretion system (*ssaV*) mutations by immunization of healthy volunteers. Infect. Immun..

[48] Blomfield I, Vaughn V, Rest R, Eisenstein B (1991). Allelic exchange in *Escherichia coli* using the *Bacillus subtilis sacB* gene and a temperature‐sensitive pSC101 replicon. Mol. Microbiol..

[49] Hitchcock PJ, Brown TM (1983). Morphological heterogeneity among *Salmonella* lipopolysaccharide chemotypes in silver-stained polyacrylamide gels. J. Bacteriol..

[50] Kang HY, Srinivasan J, Curtiss R (2002). Immune responses to recombinant pneumococcal PspA antigen delivered by live attenuated *Salmonella enterica* serovar Typhimurium vaccine. Infect. Immun..

[51] Boyd MA (2014). Serum bactericidal assays to evaluate typhoidal and nontyphoidal *Salmonella* vaccines. Clin. Vaccin. Immunol..

