# Supplementary material for: Bi-valent polysaccharides of Vi capsular and O9 O-antigen in attenuated Salmonella Typhimurium induce strong immune responses against these two antigens
Source: NPJ Vaccines. 2018 Jan 9;3:1. doi: 10.1038/s41541-017-0041-5 (PMC5760606; doi:10.1038/s41541-017-0041-5)
Supplement: Supplementary file 1 — Text S1 [file 41541_2017_41_MOESM1_ESM.docx]

## Supplemental materials and methods

## *S*. Typhimurium chromosomal modifications

Unmarked deletion and/or insertion mutations in *S*. Typhimurium were achieved by using *sacB* gene-based sucrose counter-selectable suicide vectors^1,2^. Specifically, for deletion mutation suicide vector construction, two homologous DNA fragments, upstream and downstream of the promoter, gene or operon to be deleted, were amplified using primer pairs designated D-N-1F/D-N-1R and D-N-2F/D-N-2R (N represents the name of the gene or operon to be deleted). After DNA purification, these two fragments were fused by PCR using primer pairs designated D-N-1F/D-N-2R and linked into the suicide vector pYA4278 (43). For insertion mutation suicide vector construction, the allelic promoter, gene or operon to be inserted in a directed site was amplified using primer pairs designated (G)In-I-F/ (G)In-I-R (I represents the name of the promoter, gene or operon to be inserted). The backbone of the suicide vector, which contained the homologous upstream and downstream sequence of the site to be inserted, was amplified using primer pairs designated (G)Vec-D-N-F/(G)Vec-D-N-R. All purified DNA fragments were assembled in order using Gibson Assembly ^3^, resulting in targeted promoter, gene or operon inserted into the directed site in a suicide plasmid. All allelic promoter, gene or whole-operon replacement in *S*. Typhimurium were achieved in two steps (Figure 1): First, deleted the original promoter, gene or operon and, then, inserted the target promoter, gene or operon instead. Specifically, suicide vector harbored in χ7213 was transferred to and integrated in the chromosome of parent strain by co-culturing. The successful homologous recombination event was selected on chloramphenicol agar without DAP supplement. Afterwards, the integrated suicide vector would excise from *Salmonella* chromosome due to a second homologous recombination when cultured in LB broth without chloramphenicol pressure and later counter-selected on 10% sucrose agar under 30°C. The positive *Salmonella* mutants were obtained by PCR screening and followed by other phenotype confirmations. All of the constructed mutants, either intermediates or final constructs, were routinely sequenced.

## Growth curves

The growth rates of bacterial strains were measured per 60 minutes in LB medium in a shaking incubator (37°C, 180 rpm), with an initial OD_600_ value of 0.03.

## P22 transduction studies

Evaluation of phage P22 transduction efficacy was performed as described previously ^4^. Briefly, P22HT *int* was propagated in *S*. Typhimurium S100 carrying the chromosomal-integrated chloramphenicol resistance suicide vector pSS241^4^. The recipient strains being tested were grown to an OD_600_ of 0.6 (~5 × 10^6^ CFU/ml), and 10 µl of the diluted phage (1 × 10^8^ PFU) was mixed with 1 ml of bacteria and incubated at 37°C for 30 min. After incubation, the mixture was centrifuged and resuspended in 1 ml of PBS. A 100-µl aliquot was spread on an LB agar plate containing 25 µg/ml chloramphenicol. After the plates were incubated at 37°C overnight, colonies were counted. This experiment was repeated three times.

## Motility test

Motility tests were performed on 0.3% soft agar overlays. Briefly, LB soft plates were dried at room temperature for approximately 2 h prior to performing the assays. 6 µl of freshly grown bacteria (~5 × 10^6^ CFU) was pipetted onto the center of the plates, which were then incubated at 37°C for 6 h prior to the colony diameters (in millimeters) being measured. This experiment was repeated three times.

## Minimum inhibitory concentration (MIC) test

The MICs of deoxycholate (DOC) and polymyxin B were determined using 96-well microtiter plates. Two-fold serial dilutions of DOC (0.39–59 mg/ml) and polymyxin B (0.078–10 µg/ml) were made along the plates. Bacteria were grown to an OD_600_ of 0.6 and diluted to ~5.0×10^4^ CFU/ml in LB broth. Then, 100 µl of the diluted bacteria suspension was added to each well, followed by overnight incubation at 37°C. The optical density of each well was determined using an iMark^TM^ microplate reader (Bio-Rad). The threshold of inhibition was 0.1 at OD_600_. This assay was repeated three times.

## Attachment and invasion assay

Human epithelial type 2 (Hep-2) cells (ATCC strain CCL-6) were used to perform the bacterial attachment and invasion assays as described previously ^5^. Both assays were repeated three times in triplicate.

## Colonization in mice

To evaluate colonization, three mice per group were orally inoculated with 20 μl of BSG containing 1 × 10^9^ CFU bacteria. On days 4 and 8 post-inoculation, Peyer's patches, spleen and liver samples were collected. Samples were homogenized, and dilutions were plated onto MacConkey and LB agar to determine viable counts.

1 Blomfield, I., Vaughn, V., Rest, R. & Eisenstein, B. Allelic exchange in *Escherichia coli* using the *Bacillus subtilis* *sacB* gene and a temperature‐sensitive pSC101 replicon. *Mol Microbiol* **5**, 1447-1457 (1991).

2 Nakayama, K., Kelly, S. M. & Curtiss, R. Construction of an Asd+ expression-cloning vector: stable maintenance and high level expression of cloned genes in a *Salmonella* vaccine strain. *Nat Biotechnol* **6**, 693-697 (1988).

3 Gibson, D. G. *et al.* Enzymatic assembly of DNA molecules up to several hundred kilobases. *Nat Methods* **6**, 343-345 (2009).

4 Kong, Q., Liu, Q., Roland, K. L. & Curtiss, R., 3rd. Regulated delayed expression of rfaH in an attenuated *Salmonella enterica* serovar *typhimurium* vaccine enhances immunogenicity of outer membrane proteins and a heterologous antigen. *Infect Immun* **77**, 5572-5582, doi:10.1128/IAI.00831-09 (2009).

5 Kong, Q. *et al.* Effect of deletion of genes involved in lipopolysaccharide core and O-antigen synthesis on virulence and immunogenicity of *Salmonella enterica* serovar *typhimurium*. *Infect Immun* **79**, 4227-4239, doi:10.1128/IAI.05398-11 (2011).
