# Supplementary material for: Bi-valent polysaccharides of Vi capsular and O9 O-antigen in attenuated Salmonella Typhimurium induce strong immune responses against these two antigens
Source: NPJ Vaccines. 2018 Jan 9;3:1. doi: 10.1038/s41541-017-0041-5 (PMC5760606; doi:10.1038/s41541-017-0041-5)
Supplement: Supplementary file 2 — Table S1 [file 41541_2017_41_MOESM2_ESM.docx]

| Primer name | Sequence 5'-3' |
| --- | --- |
| D-abe-1F | gtggtctggctcagttgg |
| D-abe-1R | catgaaaatccagatagaataaattgaattagaaattcaaaccaaag |
| D-abe-2F | atttattctatctggattttcatgatcttttaataaat |
| D-abe-2R | gtttttggctctgcattctgatat |
| (G)Vec-D-abe-F | actagttctatatgaatcgttaacaaattagtcgcgttatg |
| (G)Vec-D-abe-R | ttcatcgcaatcaccagatagaataaattgaattagaaattc |
| (G)(prt-tyv)-F | tttattctatctggtgattgcgatgaaaattctaataatgggagcg |
| (G)(prt-tyv)-R | taatttgttaacgattcatatagaactagtccaatcatac |
| D-ca_operon-1F | gttcataagaggtgtcgaagtgg |
| D-ca_operon-1R | gcgggtttgttagtaccgctatttttacgaaaattcc |
| D-ca_operon-2F | aaaatagcggtactaacaaacccgccattctatatttttaag |
| D-ca_operon-2R | caagttcgataacgattaacgttacc |
| (G)Vec-D-ca-F | gtttgcagaatttaacaaacccgccattctatatttttaag |
| (G)Vec-D-ca-R | taccagggggatcgtaccgctatttttacgaaaattcctgg |
| (G)In-viaB-F1 | ggcgggtttgttaaattctgcaaaccagccctgtaccatc |
| (G)In-viaB-R1 | tgatttttttcatttaacgaggctgagaggcaatgg |
| (G)In-viaB-F2 | cagcctcgttaaatgaaaaaaatcatcatattactaacgacatttttc |
| (G)In-viaB-R2 | aaatagcggtacgatccccctggtagattaaagctgc |
| D-PtviA-1F | taaggtattcatttccgaagcagtc |
| D-PtviA-1R | atgaaacctcatctagaaaatcgaatgataaattatgctaaattg |
| D-PtviA-2F | tcgattttctagatgaggtttcatcatttctggcctc |
| D-PtviA-2R | cactttccgaaaggaaatatttcatc |
| (G)Vec-D-PtviA-F | aaggaaaagcattatgaggtttcatcatttctggc |
| (G)Vec-D-PtviA-R | atccctgccaggctagaaaatcgaatgataaattatgctaaattg |
| (G)In-PssaG-F | ttcgattttctagcctggcagggattggccttgc |
| (G)In-PssaG-R | tgaaacctcataatgcttttccttaaaataaatacatcgtaag |
| D-vexE-1F | aatggtgaatggtttggatcagaac |
| D-vexE-1R | attaaatcgcttatatatttccttactcagccttattcacg |
| D-vexE-2F | aaggaaatatataagcgatttaattgcggtagatgaaaatttg |
| D-vexE-2R | ttgttttcgccagcatgaattactc |
| D-(vexA-vexE)-1F | gatggtgatttacgcgctgaag |
| D-(vexA-vexE)-1R | ttaaatcgcttattaacgaggctgagaggcaatg |
| D-(vexA-vexE)-2F | cagcctcgttaataagcgatttaattgcggtagatg |
| D-(vexA-vexE)-2R | cgccagcatgaattactctgc |

**Table S1. Primers used in this work**
