# Supplementary material for: Bi-valent polysaccharides of Vi capsular and O9 O-antigen in attenuated Salmonella Typhimurium induce strong immune responses against these two antigens
Source: NPJ Vaccines. 2018 Jan 9;3:1. doi: 10.1038/s41541-017-0041-5 (PMC5760606; doi:10.1038/s41541-017-0041-5)
Supplement: Supplementary file 3 — Table S2 [file 41541_2017_41_MOESM3_ESM.docx]

| Strain^a^ | No. of P22 transductants^b^ | MIC | | Swimming motility(mm) ^d^ | LD_50_(CFU) |
| --- | --- | --- | --- | --- | --- |
|  |  | DOC(mg/ml)^c^ | Polymyxin B(μg/ml) |  |  |
| S1137 (O4, Vi^+^, ΔP_tviA_::P_ssaG_) | 369±47 | 25 | 0.625 | 39.59 ± 0.9218 | 0.83 × 10^8^ |
| S1151 (O9, Vi^+^, ΔP_tviA_::P_ssaG_) | 374±33 | 25 | 0.625 | 41.36 ± 0.5758 | 1.05 ×10^8^ |
| S1159 (O9, Vi^+^, ΔP_tviA_::P_ssaG_ Δ*vexE*) | 442±56 | 25 | 0.625 | 34.01 ± 2.057 | 1.35 ×10^8^ |
| S1160 (O9, Vi^+^, ΔP_tviA_::P_ssaG_ Δ*vexA-E*) | 303±32 | 25 | 0.625 | 24.45 ± 1.625* | >10^8^ |
| Wild-type S100 | 487±64 | 25 | 1.25 | 41.43 ± 0.1298 | 1.59x10^5^ |

**Table S2. Transduction efficiencies, MICs, swimming motility and virulence of wild-type *S.* Typhimurium and its derivatives**

^a^ Only the immunodominant O-serotype was shown.

^b^ The phage lysate used for transduction was grown on a chloramphenicol-resistant strain. Transduction was performed as described in Materials and Methods. The results reflect the numbers of chloramphenicol-resistant colonies obtained after transduction (means ± SD).

^c^ DOC, deoxycholate.

^d^ The average diameter in millimeters (means ± SD). Significant differences was indicated as “*”, S1160 versus S100, using GraphPad Prism. *P* < 0.05.
