# Supplementary material for: Bi-valent polysaccharides of Vi capsular and O9 O-antigen in attenuated Salmonella Typhimurium induce strong immune responses against these two antigens
Source: NPJ Vaccines. 2018 Jan 9;3:1. doi: 10.1038/s41541-017-0041-5 (PMC5760606; doi:10.1038/s41541-017-0041-5)
Supplement: Supplementary file 4 — Supplementary figures [file 41541_2017_41_MOESM4_ESM.docx]

## Supplementary Figures


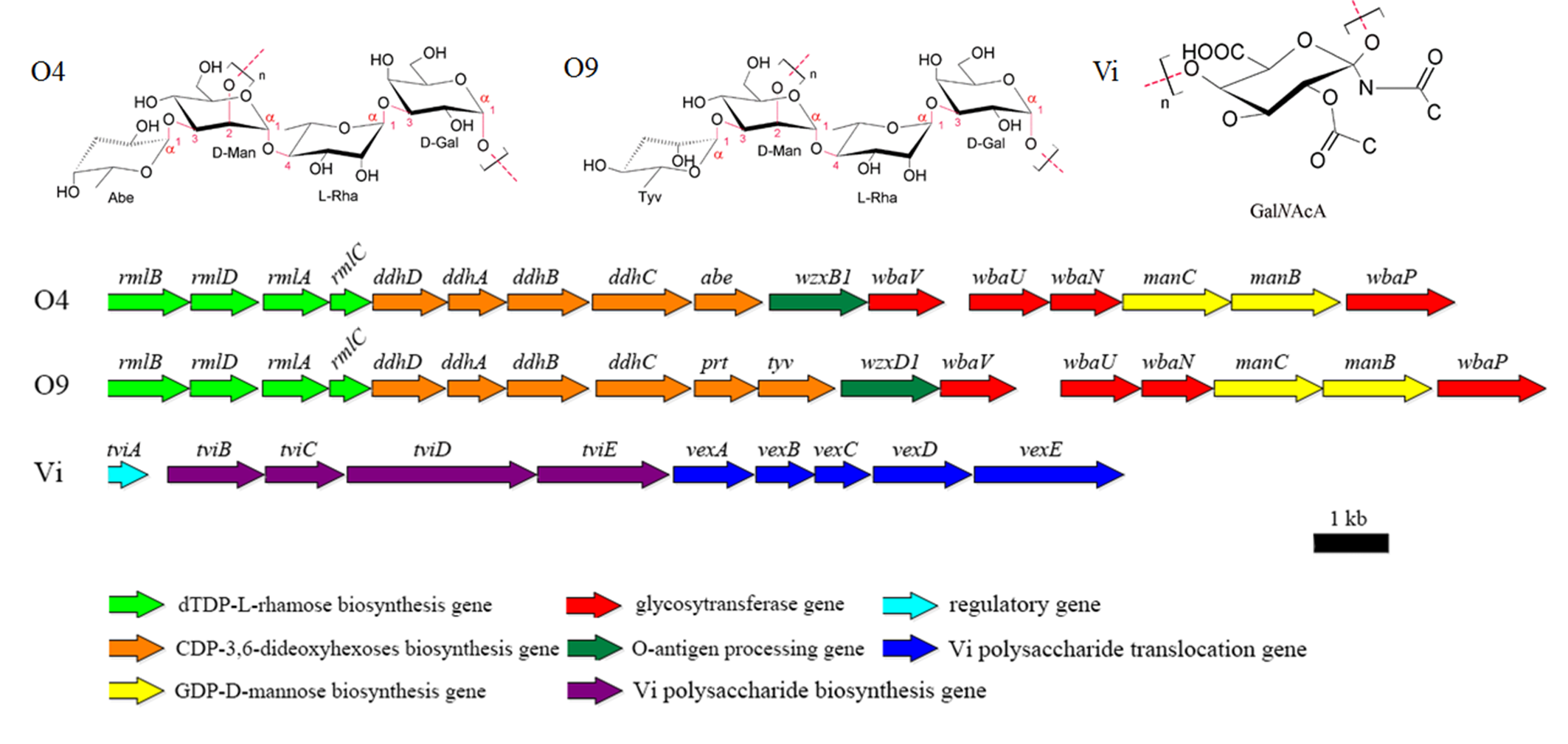


## Supplementary Figure S1. Chemical structure and gene cluster of the O4 and O9 O-polysaccharides and Vi capsular.

The chemical structures of the sugar components and glycosidic linkages within the O-units of *S.* Typhimurium, *S.* Enteritidis and Vi monomer are shown at the top. The immunodominant O-serotype factor for each serotype is indicated next to the illustrations of the O-antigen gene clusters of *S.* Typhimurium (B1, O4) and *S.* Enteritidis (D1, O9). The *viaB* locus of *S*. Typhi is shown on the bottom. Genes are color-coded indicating biosynthetic pathways, and the arrows represented the location and orientation of the genes. Sugar abbreviations: Abe, abequose; Tyv, tyvelose; L-Rha, L-rhamnose; D-Man, D-mannose; D-Gal, D-galactose; Gal*N*AcA: α-1,4-linked *N*-acetylgalactosaminuronate. Diagrams are drawn to scale.


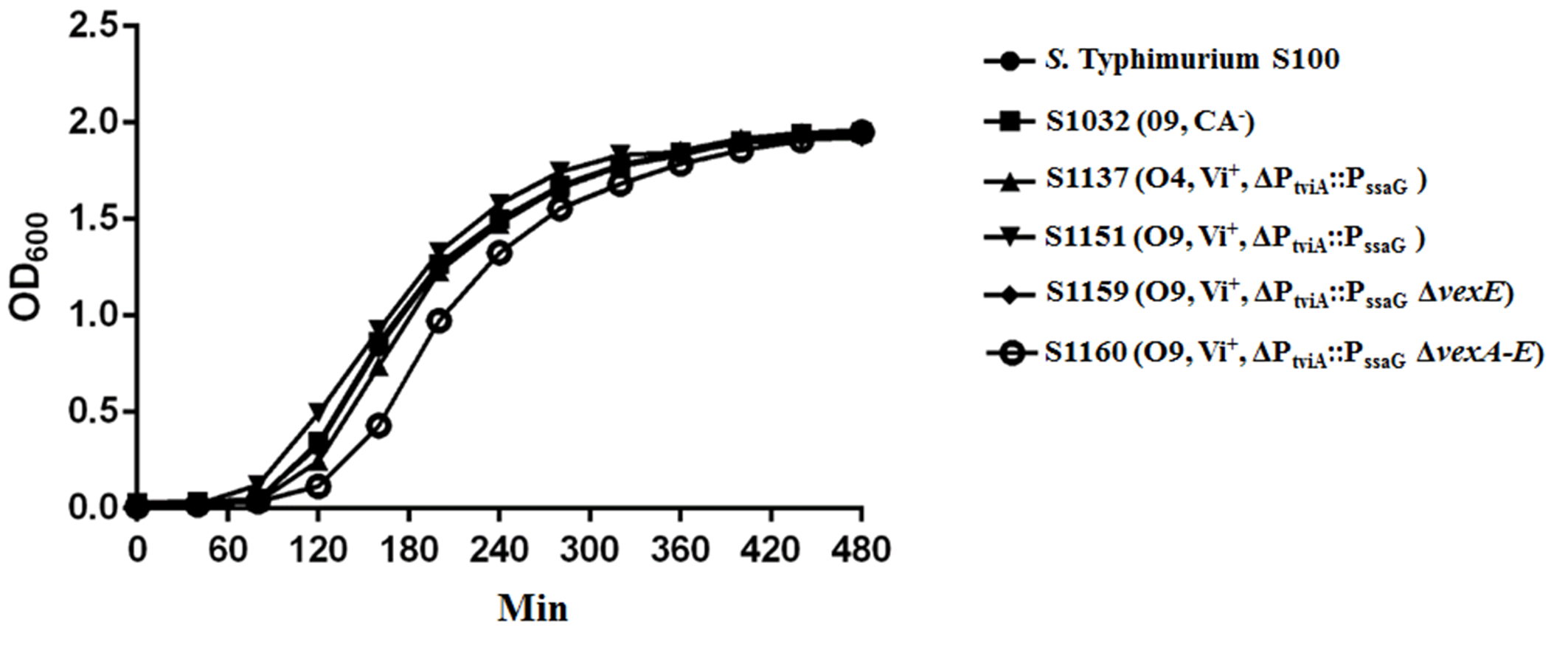


## Supplementary Figure S2. Growth curves of the *S.* Typhimurium mutants.

The *in vitro* growth rates of *S.* Typhimurium wild-type S100 and its derivatives were determined by measuring the optical density at OD_600_ at multiple time points.


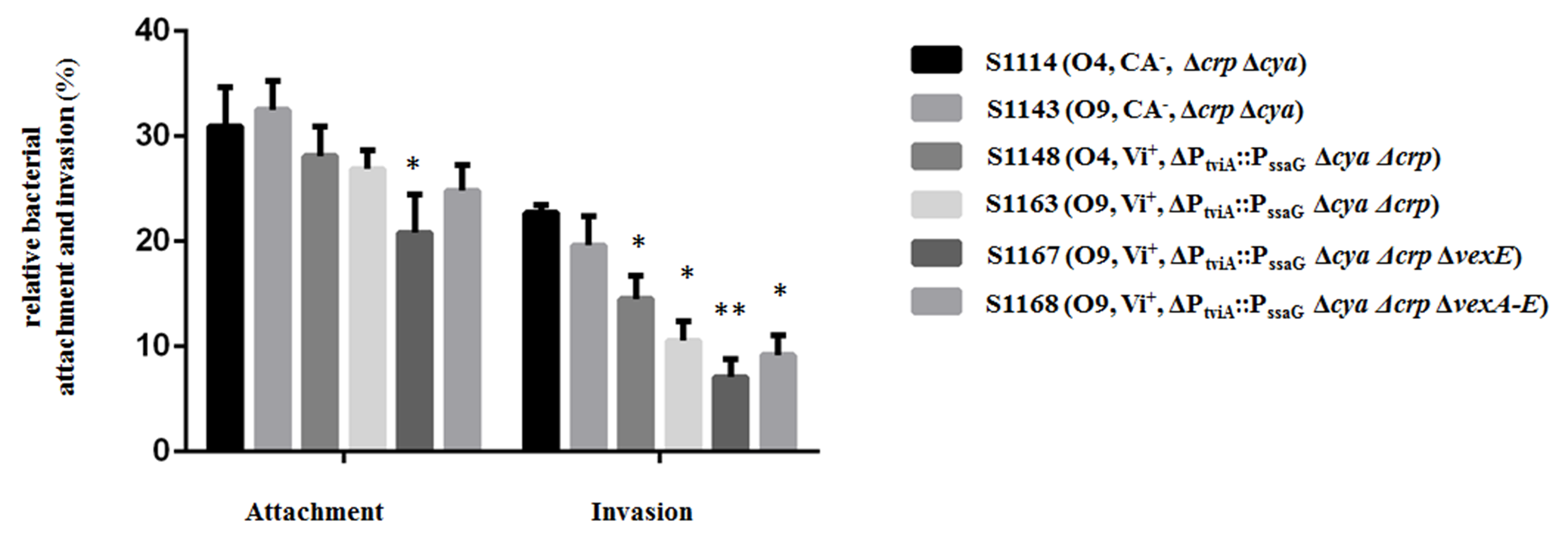


## Supplementary Figure S3. Attachment and invasion assays in Hep-2 cells.

The percentage of attachment and invasion by each *S*. Typhimurium mutant was evaluated as described in the supplementary Materials and Methods. *S*. Typhimurium cells were added to each well at a multiplicity of infection of 10:1. Compared with that of S1114, the attachment rate of S1167 and the invasion rates of 1148, S1163, S1167 and S1168 were significantly lower (*, *P*<0.05; **, *P*<0.01). Error bars represent the standard error of the means, calculated by GraphPad Prism.


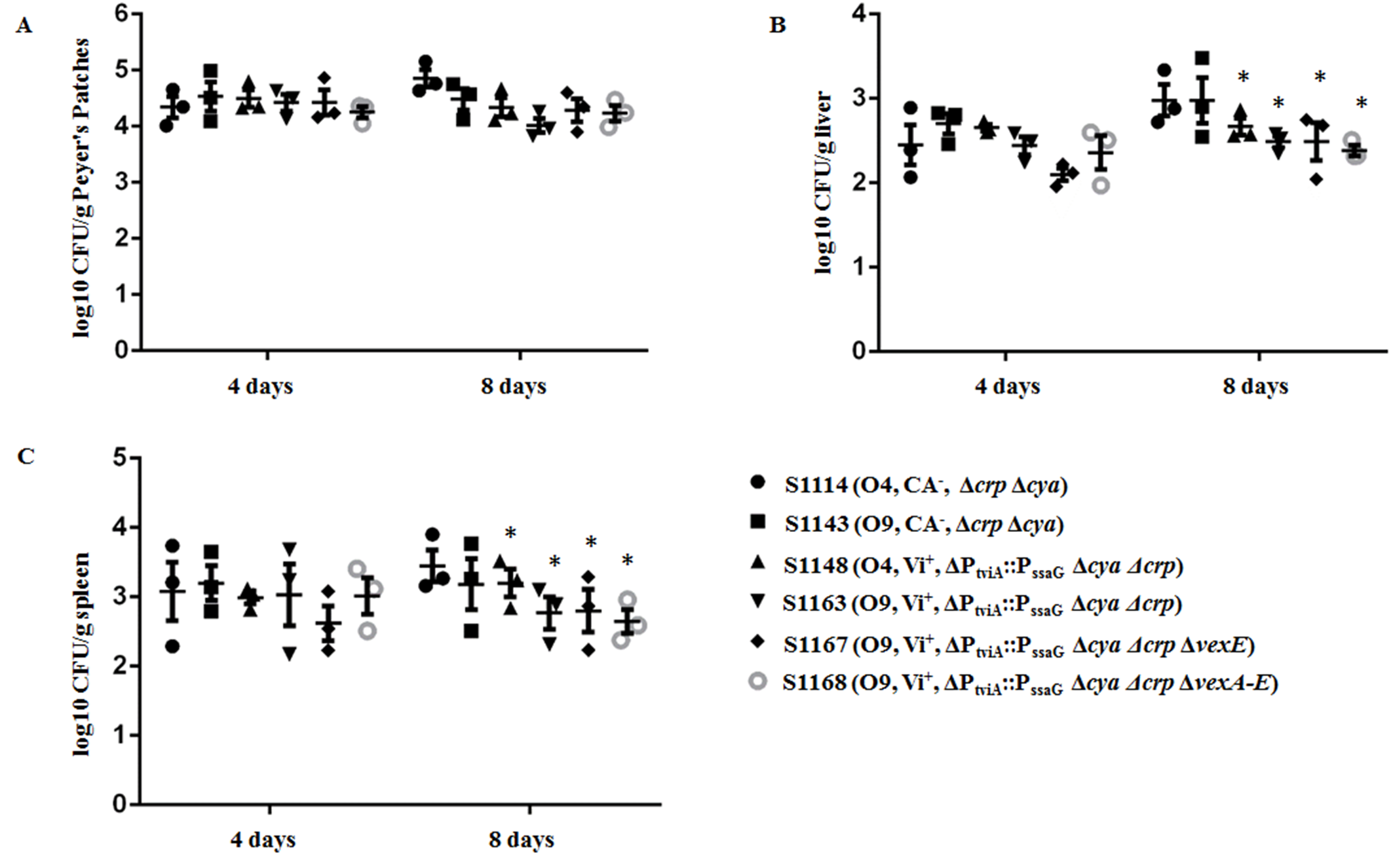


## Supplementary Figure S4. Colonization of murine Peyer's patches, livers and spleens by live attenuated *S.* Typhimurium vaccines

Colonization of mouse Peyer's patches (A), livers (B) and spleens (C) 4 and 8 days post-inoculation were shown. Strains with significantly lower rates of liver and spleen bacterial colonization compared to those of S1114 were indicated with an asterisk (*, *P*<0.05). The horizontal lines represent the means, and error bars represent the standard errors of the means.


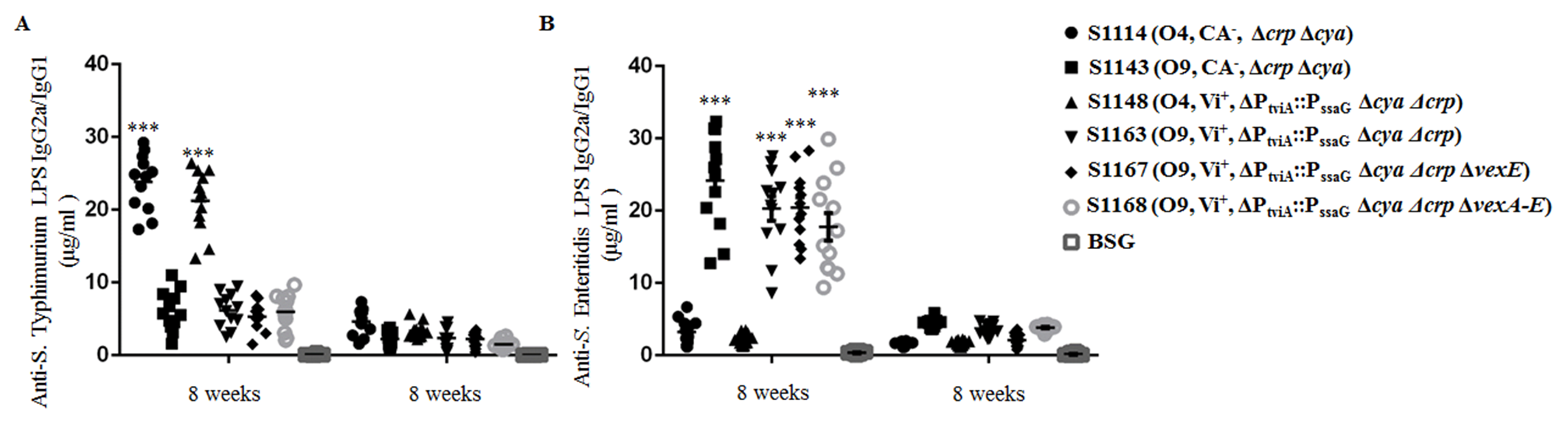


## Supplementary Figure S5. Serum IgG1 and IgG2a responses in immunized mice.

Serum responses against the LPS of *S.* Typhimurium (A) and *S.* Enteritidis (B) were determined by ELISA. The concentrations of the IgG1 and IgG2a subclasses in sera from mice vaccinated with the indicated strains are shown. A significantly higher level of IgG2a specific to the *S*. Typhimurium LPS compared to IgG1 was observed in S1114 and S1148 (***, *P*<0.001). A significantly higher level of IgG2a specific to the *S*. Enteritis LPS compared to IgG1 was observed in S1143, S1163, S1167 and S1168 (***, *P*<0.001). The antibody concentrations were calculated using a standard curve. All of the measured sample concentrations were within the standard curve range. The error bars represent the standard errors of the means.


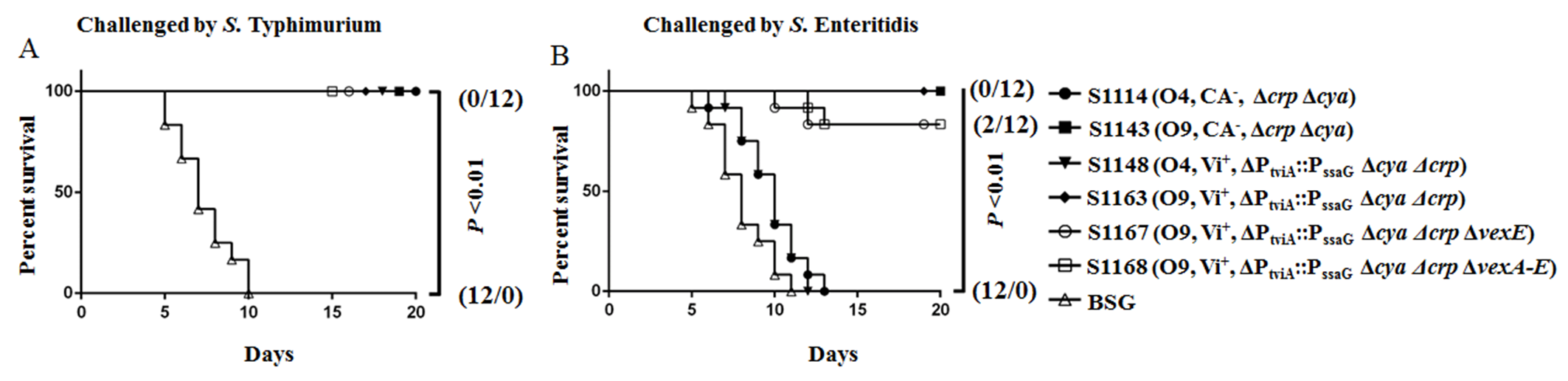


## Supplementary Figure S6. Survival curves after oral challenge by wild-type virulent *Salmonella*

Nine weeks after primary immunization, twelve BALB/c mice from each group were challenged with approximately 100 times the LD_50_ of the wild-type virulent *S.* Typhimurium (A) and *S.* Enteritidis (B). Curve comparisons were calculated using GraphPad Prism by comparing two groups by the log-rank (Mantel-Cox) test for all the marked groups versus the BSG group. *P* values were indicated.
